# High-value crops’ embedded groundnut-based production systems vis-à-vis system-mode integrated nutrient management: long-term impacts on system productivity, system profitability, and soil bio-fertility indicators in semi-arid climate

**DOI:** 10.3389/fpls.2023.1298946

**Published:** 2024-01-04

**Authors:** Ram Swaroop Bana, Anil K. Choudhary, Ravi C. Nirmal, Bhola Ram Kuri, Seema Sangwan, Samarth Godara, Ruchi Bansal, Deepak Singh, D.S. Rana

**Affiliations:** ^1^ Division of Agronomy, ICAR–Indian Agricultural Research Institute, New Delhi, India; ^2^ Division of Crop Production, ICAR-Central Potato Research Institute, Shimla, Himachal Pradesh, India; ^3^ Krishi Vigyan Kendra, Pali, ICAR-Central Arid Zone Research Institute, Jodhpur, India; ^4^ Division of Computer Applications, ICAR–Indian Agricultural Statistics Research Institute, New Delhi, India; ^5^ Division of Germplasm Evaluation, ICAR–National Bureau of Plant Genetic Resources, New Delhi, India

**Keywords:** groundnut-based cropping systems, nutrient management, organic manures, system-productivity, high-value crops, soil bio-fertility

## Abstract

The current study identified two new climate-resilient groundnut-based cropping systems (GBCSs), *viz*., groundnut–fenugreek cropping system (GFCS) and groundnut–marigold cropping system (GMCS), with appropriate system-mode bio-compost embedded nutrient management schedules (SBINMSs) for semi-arid South Asia. This 5-year field study revealed that the GMCS along with leaf compost (LC) + 50% recommended dose of fertilizers (RDF_50_) in wet-season crop (groundnut) and 100% RDF (RDF_100_) in winter-season crop (marigold) exhibited the highest system productivity (5.13–5.99 t/ha), system profits (US$ 1,767–2,688/ha), and soil fertility (available NPK). Among SBINMSs, the application of 5 t/ha leaf and cow dung mixture compost (LCMC) with RDF_50_ showed the highest increase (0.41%) in soil organic carbon (SOC) followed by LC at 5 t/ha with RDF_50_ and RDF_100_. Legume–legume rotation (GFCS) had significantly higher soil microbial biomass carbon (SMBC) and soil microbial biomass nitrogen (SMBN) than legume–non-legume rotations (groundnut–wheat cropping system (GWCS) and GMCS). Among SBINMSs, the highest SMBC (201 µg/g dry soil) and SMBN (27.9 µg/g dry soil) were obtained when LCMC+RDF_50_ was applied to groundnut. The SMBC : SMBN ratio was the highest in the GWCS. LC+RDF_50_ exhibited the highest SMBC : SOC ratio (51.6). The largest increase in soil enzymatic activities was observed under LCMC+RDF_50_. Overall, the GMCS with LC+RDF_50_ in the wet season and RDF_100_ in the winter season proved highly productive and remunerative with better soil bio-fertility. SBINMSs saved chemical fertilizers by ~25%’ in addition to enhanced system productivity and system profits across GBCSs in semi-arid regions of South Asia. Future research needs to focus on studying the potential of diversified production systems on water and environmental footprints, carbon dynamics, and energy productivity under semi-arid ecologies.

## Introduction

Due to the burgeoning population, the pressure on natural resources and soil health is on a constant rise, specifically in densely populated South Asia, which is dominated by semi-arid agro-ecology. Consequently, a plateau has been witnessed in the productivity levels of major crops and food systems of this economically, ecologically, and demographically fragile region (Nath et al., 2017; Rajpoot et al., 2021). Groundnut (*Arachis hypogaea* L.) assumes a major role in the agrarian and agro-industrial economy of South Asia, which is a major legume oilseed crop rich in both protein and oils (IOPEP, 2017; Heba et al., 2021). Hence, the declining productivity and profitability of groundnut-based cropping systems (GBCSs), especially the conventionally grown groundnut–wheat (*Triticum aestivum* L.) cropping system (GWCS) in addition to deteriorating soil health, have alarmingly threatened the agricultural sustainability of GBCSs vis-à-vis the oil supply–demand scenario in the region. Globally, groundnut is grown in an area of ~32.7 million ha (m ha) with a production of 53.9 million tons (mt) and productivity of 1,648 kg/ha (FAOSTAT, 2023). In contrast, India, the second largest groundnut producer, grows it in ~5.97 m ha area with a production of 10.2 mt and 1,716 kg/ha yield (FAOSTAT, 2023). India exported about 514,164 metric tons (MT) of groundnuts to the world, valued at US$ 629 million during 2021–2022, mainly for edible oil production and its by-product cake as protein-rich animal feed (APEDA, 2023). However, the prolonged dominance of single crop-based groundnut systems, unbalanced use of fertilizers, and reduced use of organic manures have acutely aggravated the production vulnerabilities in the region. To sustain soil health for achieving food and nutritional security, on a long-term basis, diversification of existing production systems with legumes and high-value crops and judicious nutrient management are being advocated (Ambast et al., 2006; Varatharajan et al., 2022). Among the major groundnut-based production systems, the conventional GWCS is a predominant system, particularly under irrigated semi-arid ecologies of South Asia (Heba et al., 2016; Heba et al., 2021). Nonetheless, several reports have indicated productivity stagnation of this system, chiefly owing to deterioration of soil organic carbon (SOC) levels, multi-nutrient deficiencies, multiple pest and disease infestations, and concerns about the timely and cost-effective availability of chemical fertilizers (Noman et al., 2016; Choudhary et al., 2020). We hypothesized that among the diversification options of the GWCS, the high-value crops’ embedded groundnut–fenugreek (*Trigonella foenum-graecum* L.) cropping system (GFCS) and groundnut–marigold (*Tagetes erecta* L.) cropping system (GMCS) could be more remunerative, climate-resilient, and sustainable, owing to their greater market demands, lower nutrient and water requirements, and contrasting crop-growth nature in semi-arid agro-ecologies.

Diversification of the existing GWCS vis-à-vis an effective nutrient management strategy for the diversification alternatives is equally necessary to enhance system productivity and soil health. In the recent past, escalating synthetic fertilizers’ prices have emerged as a serious concern. Further, their injudicious applications have caused harmful effects on soil and environmental health and have resulted in groundwater pollution (Sharma and Singhvi, 2017). Hence, there is an urgent need to incorporate numerous organic nutrient sources for proficient nutrient management (Sreedevi et al., 2013; Choudhary and Rahi, 2018). A judicious amalgamation of chemical fertilizers and organic nutrient sources has been well-known and strongly advocated for yield and soil fertility gains while minimizing production costs and environmental footprints (Sharma et al., 2019; Selim, 2020; Harish et al., 2022b). Additionally, the preparation of organics using diverse organic wastes not only promotes appropriate organic waste management but also supports trade-offs balancing between soil properties, crop quality, and animal health (Doran and Zeiss, 2000; Doran, 2002). Moreover, the inclusion of organics under nutrient management programs ensures a smooth supply of micronutrients as well as major nutrients (Bana et al., 2018; Rajpoot et al., 2021). The usage of organics also bestows a favorable micro-environment for crop growth and development, principally by improving soil properties (Bana et al., 2012; Bana et al., 2016; Bana et al., 2018; Bhupenchandra et al., 2022a).

Further, soil biological functions, the vital subset of soil health, are characterized by diverse indicators including soil microbial biomass carbon (SMBC), soil microbial biomass nitrogen (SMBN), and activities of soil microbial enzymes (dehydrogenase, protease, acid, and alkaline phosphatase) (Van Bruggen and Semenov, 2000; Singh et al., 2020; Singh et al., 2021; Singh et al., 2022a; Singh et al., 2022b). Owing to the potential of soil enzymes in plant nutrient availability via nutrient transformations, their activity in the soil is considered the most potent ecological indicator for a soil health assessment (Karlen et al., 1997; Alkorta et al., 2003; Singh et al., 2022b). Moreover, the soil biological functions are enormously dynamic and extremely sensitive to agronomic management practices like cropping system diversification (Yusuf et al., 2009), nutrient management (Liu et al., 2010; Bana et al., 2015; Ghosh et al., 2017), residue recycling, and crop establishment methods (Srinivasarao et al., 2013; Naragund et al., 2020; Harish et al., 2022a). Therefore, it is crucial to have deeper insights and understanding of the effect of diverse cropping systems and contrasting nutrient management practices on SMBC, SMBN, and soil microbial enzymatic activities for designing more appropriate and economically and ecologically sound management practices for the long-term sustainability of food systems (Bhupenchandra et al., 2022a).

Relative comparisons of diverse cropping systems concerning yield, economics, and soil fertility have been documented well across the globe and specifically in South Asia (Das et al., 2013; Heba et al., 2016; Heba et al., 2021; Rajpoot et al., 2021), but there is a lack of understanding about the diversification effects on soil biological functions (Bana et al., 2023). A knowledge gap exists on the inclusion of fenugreek and marigold in rotation with groundnut and its effect on system productivity, profitability, and soil health. Similarly, it has been established that plant nutrition protocols considerably influence soil fertility and its physical activity in addition to its microbial activity. Constant usage of chemical fertilizers, however, differently affects the soil systems as compared to the integrated application of organics and synthetic fertilizers (Tamilselvi et al., 2017). The composition and amount of applied organic manures also differentially affect the soil microbial diversity, abundance, and soil enzymatic activity (Chinnadurai et al., 2014; Kumar et al., 2017; Nath et al., 2017). Hence, the amalgamation of organic manures, prepared using locally available farm resources with chemical fertilization, was hypothesized to influence soil health (both chemical and microbial) and system productivity positively. Furthermore, medium- or long-term effects of chemical fertilizers and the integrated application of organic and inorganic nutrients need thorough investigation. Considering the above, a medium-term study was undertaken to design diversification options for the GWCS for improved yield and soil health and to assess the impact of different practices on system productivity, farm profits, and soil biological and chemical properties.

## Materials and methods

### Experimental site

**Figure 1:**
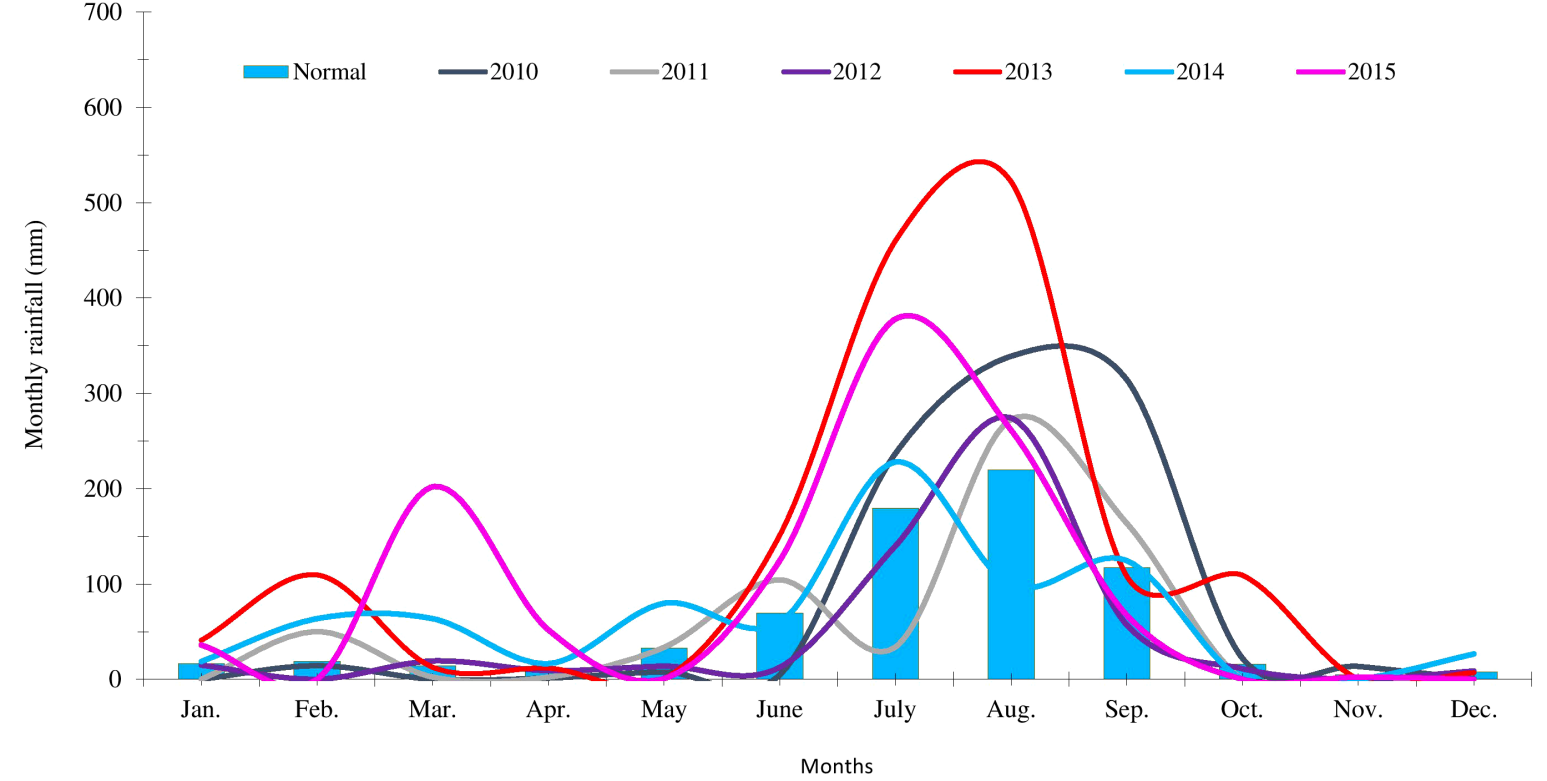
Mean monthly rainfall received during the period of experimentation.

The fixed plot field experiment was performed for five consecutive years from 2010–2011 to 2014–2015 at ICAR–Indian Agricultural Research Institute (IARI), New Delhi, India (latitude 28°4′N; longitude 77°12′E; altitude 228.6 m). Delhi falls under a semi-arid climatic zone where ~70%–80% of the annual rainfall (652 mm) is received from July to September and the rest during winter (Bana et al., 2016). The mean monthly rainfall received during the period of experimentation is presented in **Figure 1**. The soil of the experimental site was sandy-loam in texture (Inceptisol), slightly alkaline in reaction, poor in SOC and available N, and medium in available P and K. Detailed physico-chemical properties of experimental soil are presented in **Table 1**.

**Table 1:** Physical and chemical properties of the top 15-cm soil of the experimental field.

Particulars	Content	Method of analysis
A. Soil physical analysis		
Sand (%)	60.9	Modified hydrometer method (Bouyoucos, 1962)
Silt (%)	13.2	
Clay (%)	25.9	
Soil texture class	Sandy-loam	
B. Soil chemical analysis		
1. Soil organic carbon (%)	0.36	Walkley and Black method (Jackson, 1973)
2. Available N (kg/ha)	170.1	Modified Kjeldahl’s method (Jackson, 1958)
3. Available P (kg/ha)	15.9	Olsen’s method (Olsen et al., 1954)
4. Available K (kg/ha)	199.6	Flame photometer method (Jackson, 1958)
5. pH (1:2.5 soil:water)	7.7	Blackman’s Xeromatic pH meter (Jackson, 1958)
6. EC (dS/m at 25°C)	0.35	(Jackson, 1958)
7. DTPA extractable Zn (mg/kg)	0.59	(Lindsay and Norvell, 1978)
8. DTPA extractable Fe (mg/kg)	4.81	

### Treatment detail and crop management

The experiment was laid out in a split-plot design with three replications. In the main plots, three diversified cropping systems were allocated. Four nutrient management practices applied to groundnut were kept in sub-plots, whereas in sub-sub plots, two nutrient management treatments were applied to winter-season crops, hence with a total of eight system-mode bio-compost embedded nutrient management schedules (SBINMSs) under each cropping system (**Table 2**). All the treatments were kept on the same plots for 5 years under conventionally tilled conditions. Before the sowing of crops, a deep plowing using a soil turning plow, followed by three passes of a 9-tyne cultivator, was performed to prepare the field for sowing. Groundnut variety ‘GG-10’ was sown in the first week of July during all 5 years of study, at 40-cm row spacing using 100 kg/ha seed rate. Gap filling and thinning operations were performed within 20 days of sowing. The recommended dose of fertilizers (RDF) for groundnut was 25 kg N + 60 kg P_2_O_5 + _40 kg K_2_O per ha. The chemical fertilizers were placed at the time of final tillage before the sowing of groundnut. Under SBINMSs, the organic manures were applied 3 weeks before groundnut sowing. The application rate of leaf compost (LC) and leaf and cow dung mixture compost (LCMC) (containing mean N, P_2_O_5_, and K_2_O content of 0.63%, 0.31%, and 0.67% and 0.57%, 0.33%, and 0.51%, respectively) was performed using 5 t/ha composts on an oven-dry weight basis. To control weeds in groundnut, pre-emergence application of pendimethalin was performed using 0.75 kg a.i. ha^−1^ in 500 L/ha spray solution. Winter crops were grown as per the standard package of practices, recommended by ICAR-IARI, New Delhi. For wheat, the ‘HD-2967’ variety was sown using a seed drill in the second week of November at a row spacing of 25 cm. The RDF of wheat, 120 kg N + 60 kg P_2_O_5 + _60 kg K_2_O per ha, was applied as per the treatments (**Table 2**). Similarly, the RDF for fenugreek and marigold was 20 kg N + 60 kg P_2_O_5 + _60 kg K_2_O/ha and 120 kg N + 60 kg P_2_O_5 + _60 kg K_2_O/ha, respectively. For fenugreek, the ‘RMt 305’ variety was sown using a 25 kg/ha seed rate with 25-cm row spacing. Likewise, 4-week-old seedlings of the ‘Pusa Narangi Gainda’ variety of marigold were transplanted in the second week of November. The N, P, and K were applied through urea (46% N), single superphosphate (16% P_2_O_5_), and muriate of potash (60% K_2_O), respectively, as per the treatments (**Table 2**).

**Table 2:** Details of high-value crops’ embedded groundnut-based cropping systems (GBCSs) vis-à-vis their system-mode bio-compost embedded nutrient management schedules (SBINMSs).

S. no.	Cropping systems (main plot)	Treatment applied to groundnut in wet season (sub-plot)	Treatment applied to crops in winter season (sub-sub-plot)	Treatment combinations
1	C1: Groundnut–wheat cropping system [GWCS]	F1: No fertilizers in the wet season [control]	S1: 100% recommended dose of fertilizers (RDF) [F100]	C1F1S1
2			S2: 50% RDF [F50]	C1F1S2
3		F2: 100% RDF [100 RDF]	S1: 100% RDF [F100]	C1F2S1
4			S2: 50% RDF [F50]	C1F2S2
5		F3: Leaf compost (LC) at 5 t/ha + 50% RDF [LC+RDF_50_]	S1: 100% RDF [F100]	C1F3S1
6			S2: 50% RDF [F50]	C1F3S2
7		F4: Leaf and cow dung mixture compost (LCMC) at 5 t/ha + 50% RDF [LCMC+RDF_50_]	S1: 100% RDF [F100]	C1F4S1
8			S2: 50% RDF [F50]	C1F4S2
9	C2: Groundnut–fenugreek cropping system [GFCS]	F1: No fertilizers in the wet season [control]	S1: 100% RDF [F100]	C2F1S1
10			S2: 50% RDF [F50]	C2F1S2
11		F2: 100% RDF [100 RDF]	S1: 100% RDF [F100]	C2F2S1
12			S2: 50% RDF [F50]	C2F2S2
13		F3: LC at 5 t/ha + 50% RDF [LC+RDF_50_]	S1: 100% RDF [F100]	C2F3S1
14			S2: 50% RDF [F50]	C2F3S2
15		F4: LCMC at 5 t/ha + 50% RDF [LCMC+RDF_50_]	S1: 100% RDF [F100]	C2F4S1
16			S2: 50% RDF [F50]	C2F4S2
17	C3: Groundnut–marigold cropping system [GMCS]	F1: No fertilizers in wet season groundnut [control]	S1: 100% RDF [F100]	C3F1S1
18			S2: 50% RDF [F50]	C3F1S2
19		F2: 100% RDF [100 RDF]	S1: 100% RDF [F100]	C3F2S1
20			S2: 50% RDF [F50]	C3F2S2
21		F3: LC at 5 t/ha + 50% RDF [LC+RDF_50_]	S1: 100% RDF [F100]	C3F3S1
22			S2: 50% RDF [F50]	C3F3S2
23		F4: LCMC at 5 t/ha + 50% RDF [LCMC+RDF_50_]	S1: 100% RDF [F100]	C3F4S1
24			S2: 50% RDF [F50]	C3F4S2

### Soil sampling and analysis

From the fixed plots, soil samples were taken using a core auger from 0–15-cm depth instantaneously after harvest and were transferred to the laboratory for microbial analysis. Soil acid and alkaline phosphatase enzymatic activities were determined using 16 mM of para (*p*)-nitrophenyl phosphate as substrate and reported as μmol *p*-nitrophenol·g^−1^·h^−1^ (Tabatabai and Bremner, 1969). Dehydrogenase activity was determined using the rate of reduction of triphenyl tetrazolium chloride to triphenyl formazan and expressed as μg TPF·g^−1^·24 h^−1^. The chloroform-fumigation extraction method was employed for the analysis of SMBC and SMBN and expressed as µg/g of dry soil (Vance et al., 1987). The value of the efficiency of extraction of microbial biomass carbon (kEC) was 0.45 (Jenkinson and Powlson, 1976). The value of efficiency of extraction of microbial biomass nitrogen (kEN) was 0.68 (Brookes et al., 1985). The optical density at 485 nm was compared to that of the triphenyl formazan standard (Casida et al., 1964). Soil with 1% casein as the substrate was incubated for 2 h in 0.05 M tris-hydroxymethyl-aminomethane-hydrochloric acid buffer at pH 8 for protease enzyme determination. The released amino acid was analyzed through the Folin–Ciocalteu colorimetric method described by Ladd and Butler (1972) and expressed as µmol tyrosine·g^−1^ soil·h^−1^.

### Soil nutrient analysis

Soil samples from 0–15-mm depth were collected using the core sampler to assess the treatment effects on the fertility status of the soil after harvest as suggested by Rana et al. (2014). Soil samples were analyzed to study available N, P, and K through modified Kjeldahl’s method (Jackson, 1958), Olsen’s method (Olsen et al., 1954), and the flame photometer method (Jackson, 1958), respectively. Before the initiation of experiments, the extractable Zn, Mn, Fe, and Cu were determined in soil samples using DTPA (Lindsay and Norvell, 1978).

### System productivity and profitability

The yield of the crops was recorded from the net plot area and expressed in t/ha. For comparing the different production systems, the yield of winter crops was converted as groundnut equivalent yield (GEY). The GEY of winter crops was calculated using Equation 1:


(1) $$Ye=\frac{Yw}{Pg}\times Pw,$$

where Ye is GEY, Yw is the economic yield of winter crop, Pg is the market price of groundnut, and Pw is the market price of winter crop.

To compute system productivity of each production system, the following equation was used (Equation 2):


(2) Ps=Y+Ye,

where Ps is the system productivity, Y is the yield of groundnut, and Ye is the GEY of winter crops.

### Statistical analysis

The data on various parameters were statistically analyzed as per the procedure of analysis of variance (ANOVA) to determine treatment effects through Tukey’s honestly significant difference test as a *post hoc* mean separation test (*p*< 0.05) using SAS 9.1 software (SAS Institute, Cary, NC, USA). Tukey’s procedure was used where ANOVA was found significant. Correlation analyses and treatment means were compared at a 5% level of significance.

## Results and discussion

### Soil biological and chemical health

**Figure 2:**
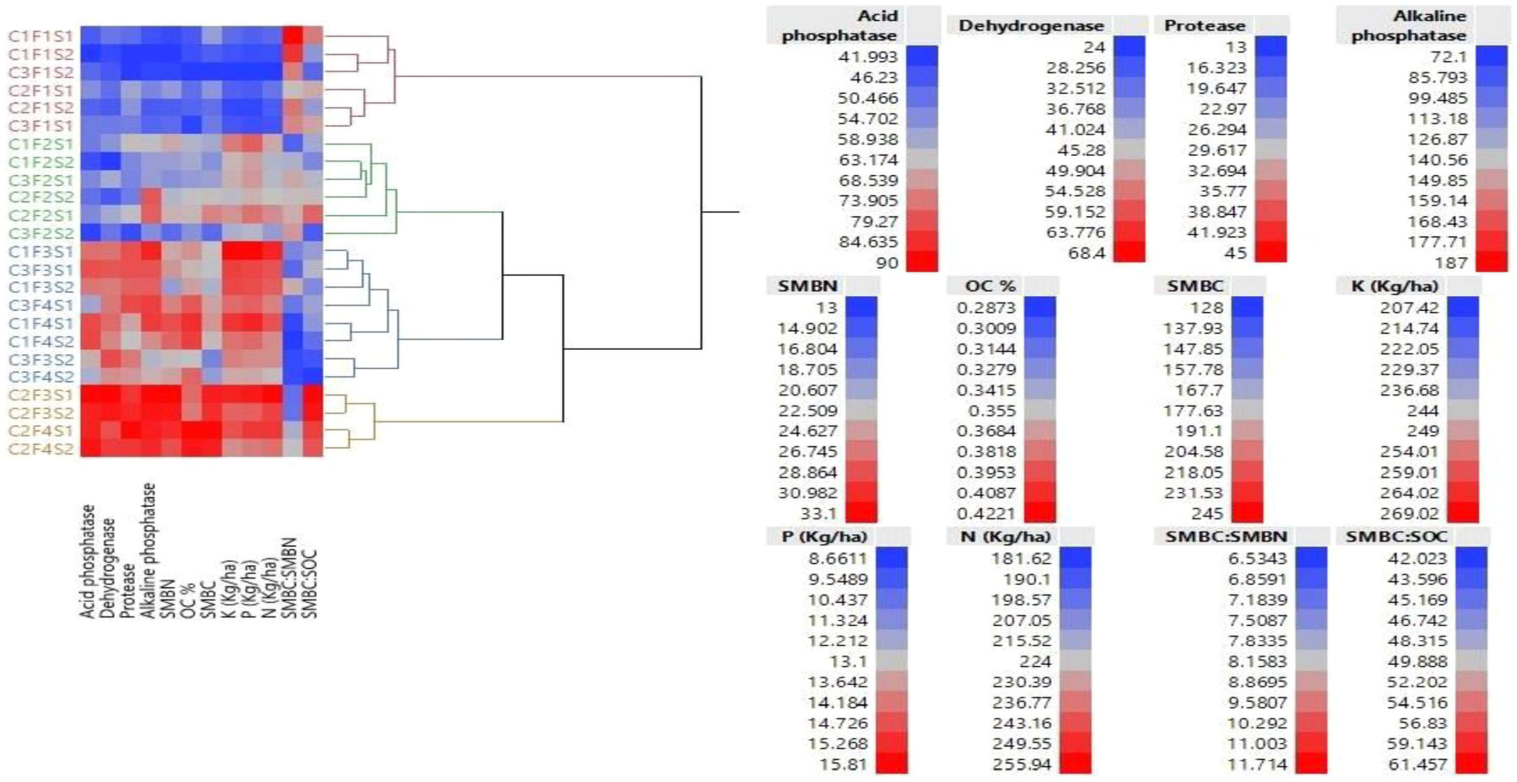
Dendrogram displays hierarchical clustering of experimental treatments based on the levels of soil bio-fertility indicators. Red color indicates higher values of health parameters, which gradually fades into blue color, indicating lower values.

Deteriorating soil health is an emerging issue in agriculture. The quality and quantity of food produced through agriculture largely depend on the status of soil health. One of the main objectives of this study was to analyze the effect of different GBCSs and SBINMSs on soil health. Soil health was assessed using the following soil health parameters: SOC, nitrogen (N), phosphorus (P), potassium (K), SMBC, SMBN, SMBC : SMBN, SMBC : SOC, acid phosphatase, alkaline phosphatase, dehydrogenase, and protease. Based on the measured levels of soil health parameters, treatments were clustered into four groups in a hierarchical manner, which is displayed in a dendrogram (**Figure 2**). The group that showed superior soil health consisted of four treatments of integrated nutrient management in the GFCS, followed by the integrated nutrient management (INM) treatments of the GWCS. Poor soil health was observed for all controls. Results for each soil health parameter are discussed below.

### Soil organic carbon

**Figure 3:**
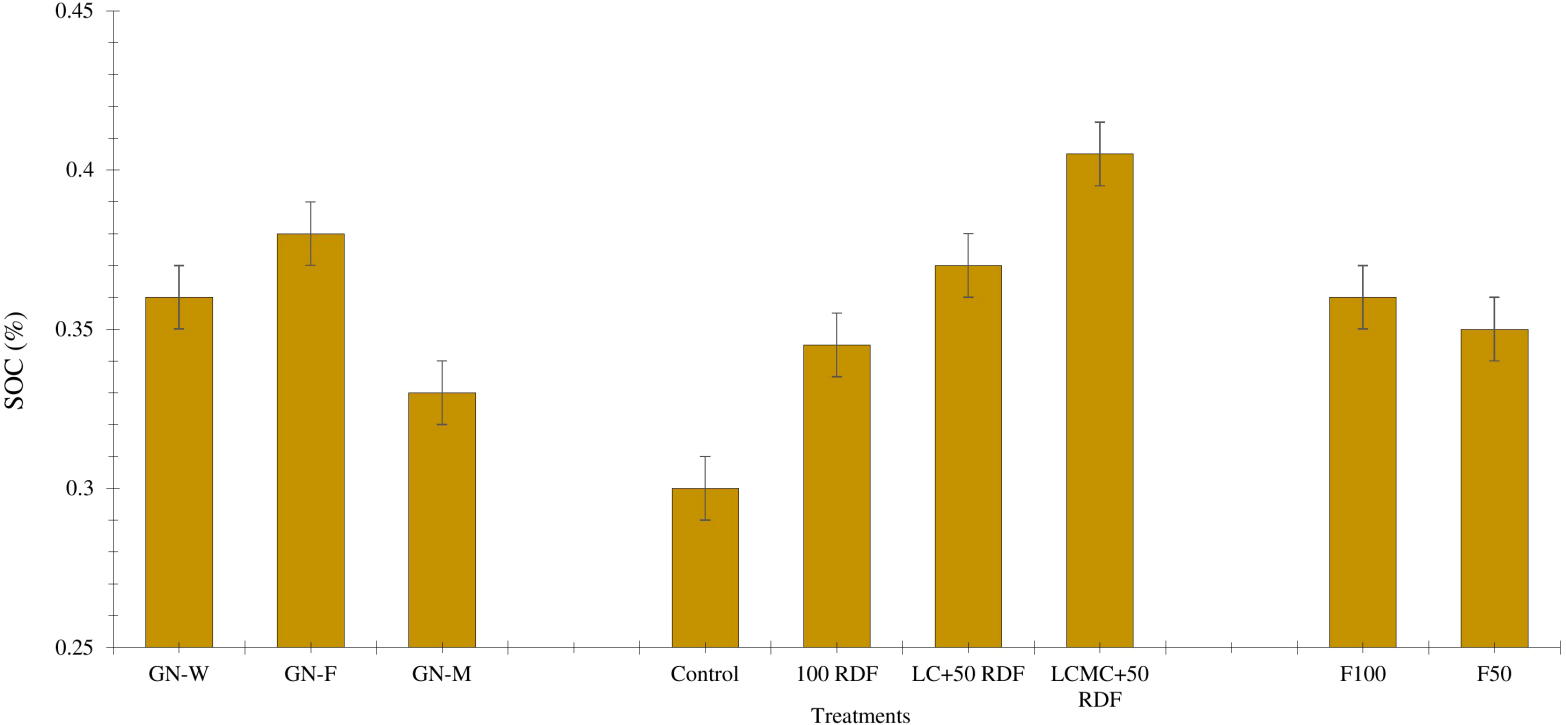
Effect of high-value crops’ embedded groundnut-based cropping systems (GBCSs) and system-mode bio-compost embedded nutrient management schedules (SBINMSs) on soil organic carbon (SOC).

Levels of SOC significantly varied among the cropping systems and nutrient management practices in wet-season crops, but they were non-significantly affected by fertilizer application in winter-season crops (**Figure 3**). The initial SOC content of the soil was 0.36% (**Table 1**). The GFCS exhibited a 0.02% increase in SOC content, whereas a decline of 0.03% in SOC was observed in the GMCS. The initial amount of SOC content was maintained as it is in the GWCS. In the long run, legumes have been observed to increase SOC (Nath et al., 2019; Choudhary et al., 2020). Among the three experimental crops, leaf-litter fall was the highest in fenugreek followed by wheat and then marigold. In addition to that, the marigold crop was uprooted after the season. Thus, nutrient and biomass recycling was less in the marigold. However, more crop residues of wheat and fenugreek remained in the field, leading to higher nutrient and biomass recycling. This might have resulted in higher levels of SOC (Saha and Ghosh, 2013). The highest decline of 0.06% in SOC was observed in control plots over initial levels of SOC. The application of 5 t/ha LCMC with 50% RDF showed the highest increase of 0.41% in SOC content, followed by LC at 5 t/ha with 50% RDF (0.37%) and 100% RDF (0.36%). The 100% RDF treatment had significantly higher SOC content (0.35%) than control (0.30%). These results indicate that an integrated approach to system-basis nutrient management through SBINMSs can efficiently increase the levels of SOC (Bana et al., 2016; Srinivasarao et al., 2021).

### Soil fertility

**Figure 4:**
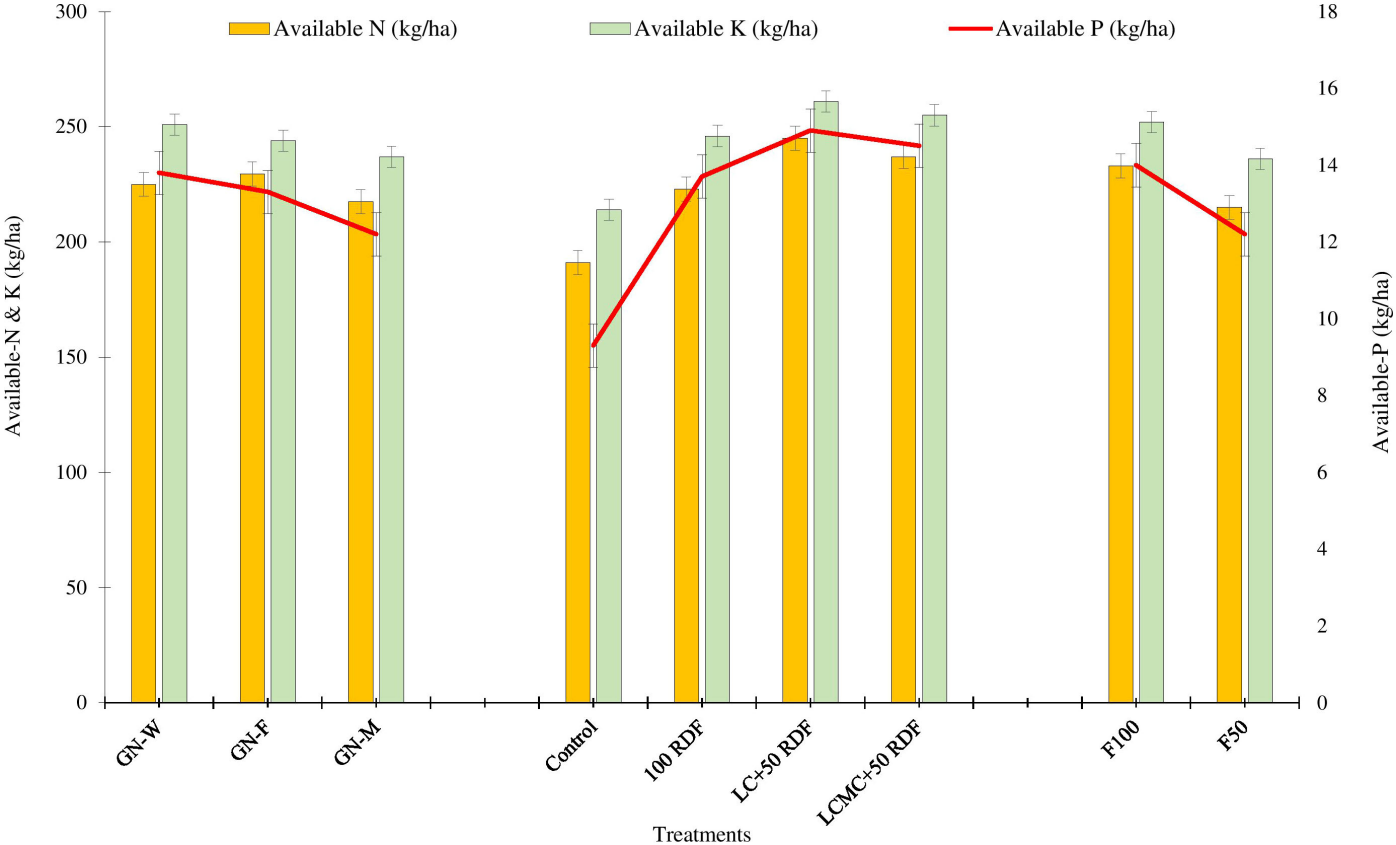
Effect of groundnut-based cropping systems (GBCSs) and system-mode bio-compost embedded nutrient management schedules (SBINMSs) on soil fertility status in terms of available NPK in the soil.

After 5 years of experimentation, significant improvement in soil fertility concerning available NPK levels under various treatments was observed (**Figure 4**). The data show that the GFCS and GWCS rotations had statistically similar effects on soil available N and P levels. Compared to these rotations, the soil fertility was slightly lower in the GMCS. This could be correlated with lower biomass recycling in the marigold as mentioned previously. Available K was found statistically similar throughout all three experimental cropping systems. Higher improvement in soil fertility was observed after the application of LC and LCMC in groundnut compared to control and chemical fertilizers. The available N content was 191 kg/ha in control, which was improved by 32 kg/ha, 54 kg/ha, and 46 kg/ha, respectively, due to the application of 100 RDF, LC+RDF_50_, and LCMC+RDF_50_. The P content was found to be significantly higher when either leaf compost (14.9 kg/ha) or mixed compost (14.5 kg/ha) was applied. With the application of LC+RDF_50_, the available P content was enhanced by 1.18 kg/ha and 5.57 kg/ha compared with 100 RDF and control, respectively. The soil K content ranged from 214 kg/ha to 261 kg/ha. The data revealed that available K was the least in control, while it increased with the addition of both inorganic and organic fertilizers. The highest K was recorded in LC+RDF_50_ whose effect was statistically similar to that of the mixed compost. Fertilizer application in winter crops also affected the soil fertility status considerably. Significantly higher soil NPK content was observed in the F100 compared with F50. Leaf compost and mixed compost contain lower nutrients but have sufficient soil organic matter (SOM), hence improving soil health (Choudhary et al., 2020). Further, the SOM increases the water-holding capacity of the soils and also makes more nutrients available to the plants (Richardville et al., 2022). It can be inferred from the results that as LC acts as more of a soil conditioner, soil retains externally applied inorganic nutrients more efficiently and improves nutrient availability to plants (Bhupenchandra et al., 2022b; Gupta et al., 2022).

### Soil microbial properties

**Figure 5:**
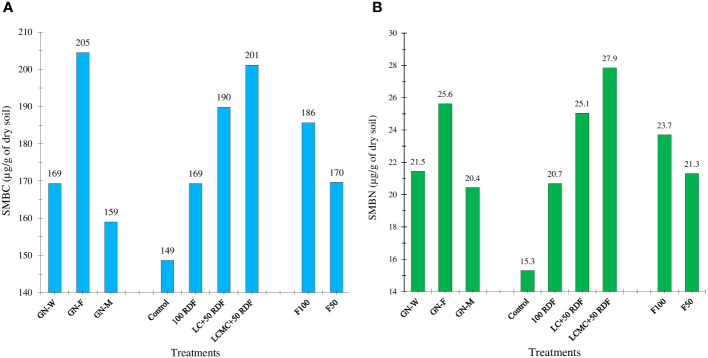
Effect of groundnut-based cropping systems (GBCSs) and system-mode bio-compost embedded nutrient management schedules (SBINMSs) on **(A)** soil microbial biomass carbon (SMBC) and **(B)** soil microbial biomass nitrogen (SMBN).

**Figure 6:**
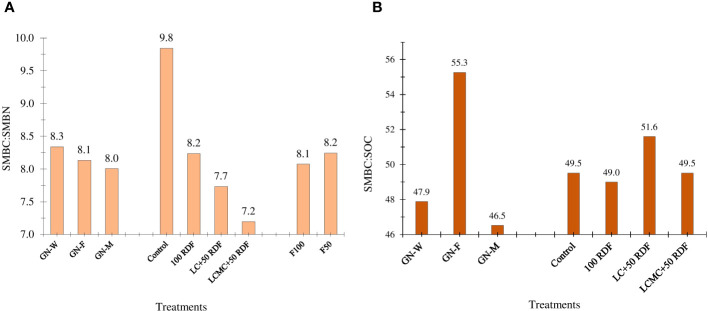
Effect of groundnut-based cropping systems (GBCSs) and system-mode bio-compost embedded nutrient management schedules (SBINMSs) on **(A)** soil microbial biomass carbon to soil microbial biomass nitrogen (SMBC : SMBN) ratio and **(B)** soil microbial biomass carbon to soil organic carbon (SMBC : SOC) ratio.

The legume–legume crop rotation (GFCS) had significantly higher levels of SMBC and SMBN compared to legume–non-legume rotations (GWCS and GMCS) (**Figure 5**). The practice of crop rotation has been observed to alter the status of soil nutrients through microbial immobilization (Balota et al., 2003; Bhupenchandra et al., 2022a; Bhupenchandra et al., 2022c). In this study, the highest SOC was noted for the GFCS. Borase et al. (2020) also demonstrated a positive correlation between SOC, SMBN, and SMBC. Among the cropping systems, the highest values of SMBC (205 µg/g of dry soil) and SMBN (25.6 µg/g of dry soil) were observed in the GFCS followed by the GWCS. The lowest values of SMBC (159 µg/g of dry soil) and SMBN (20.4 µg/g of dry soil) were recorded in the GMCS. Nutrient management strategies in groundnut had a considerable impact on SMBC and SMBN. Compared to the control and 100% RDF, a noticeable increase in both SMBC and SMBN was observed in the INM approach. The highest SMBC (201 µg/g of dry soil) and SMBN (27.9 µg/g of dry soil) were obtained when 5 t/ha LCMC+RDF_50_ was applied to groundnut. Higher microbial count in manure compost could likely be the reason for these higher values (Bana et al., 2012; Bhupenchandra et al., 2022a). Compared to F50, the F100 improved both SMBC and SMBN in the study. The legume–cereal rotation had a higher SMBC : SMBN ratio than the legume–legume and legume–marigold rotations (**Figure 6A**). The ratio of SMBC : SMBN was the highest in the GWCS (8.3), followed by the GFCS, and the least in the GMCS (8.0). The SMBC : SMBN ratio was maximum for control (9.8). In contrast, the decline in the SMBC : SMBN ratio was observed after the application of chemical fertilizers and organic manures. The plots receiving LCMC+RDF_50_ recorded the least SMBC : SMBN (7.2). The addition of N through fertilizers was reported to reduce the microbial C:N ratio of the soil (N addition effect on microbes). The application of F100 to winter crops lowered the SMBC : SMBN ratio compared with F50. The ratio of SMBC : SOC was the highest for the GFCS (55.3) and lowest for the GMCS (46.5) (**Figure 6B**). Among the nutrient treatments, LC+RDF_50_ (51.6) recorded the highest SMBC : SOC ratio, while RDF had the least SMBC : SOC ratio (49).

One of the prime indicators of healthy soil is biologically alive soil containing a healthy microbial community (Singh et al., 2021; Singh et al., 2022a; Singh et al., 2022b). Microbial activity in soil is mostly correlated with available SOM (Zhong and Cai, 2007). Microbes primarily feed on SOM and make several nutrients available to plants, which are produced as their metabolic by-products, among which carbon and nitrogen are especially of importance for plant growth (Perelo and Munch, 2005; Six et al., 2006; Bhupenchandra et al., 2022a; Bhupenchandra et al., 2022c). Microbes, while feeding on SOM, also release many biostimulants and hormones, which again help in enhancing plant growth and productivity (Bhupenchandra et al., 2022c; Rajanna et al., 2023). The interactive effect of soil microbes on other soil components improves the soil structure. The type of plant root system directly influences the growth of these micro-organisms. Leguminous plants are well known to fix atmospheric N through their root nodules. They also have been observed to increase the levels of SMBN, SMBC, and SOC in the soil in several studies (Zahran, 1999; Mayer et al., 2004; Liu et al., 2011; Wang et al., 2018; Bhupenchandra et al., 2022a). Wheat roots are fibrous and usually go 100–200 cm deep down into the soil. In contrast, fenugreek and marigold root systems are shallow and remain confined largely into or adjacent to the plow layer only (0–15 cm deep). Therefore, wheat adds more SOM and improves the soil microbial activity. Wheat root exudates such as glues, gums, and waxes help in improving the soil structure by providing adherence to soil molecules, thus increasing the stability of soil aggregates (Bana et al., 2018), which again helps in better aeration and water infiltration (Choudhary and Rahi, 2018). The cumulative effect of all these factors creates a better environment for flourishing microbial growth (Mauchline et al., 2015). Manure compost is rich in nutrients as well as in microbial count; hence, it acts as microbial inoculum for the soil (Ren et al., 2019). The mineral-based inorganic fertilizers do not help in significantly increasing the microbial activity of the soil, compared to organic fertilizers (Lazcano et al., 2013; Kumar et al., 2021; Kumar et al., 2022).

### Soil enzymatic activities

**Figure 7:**
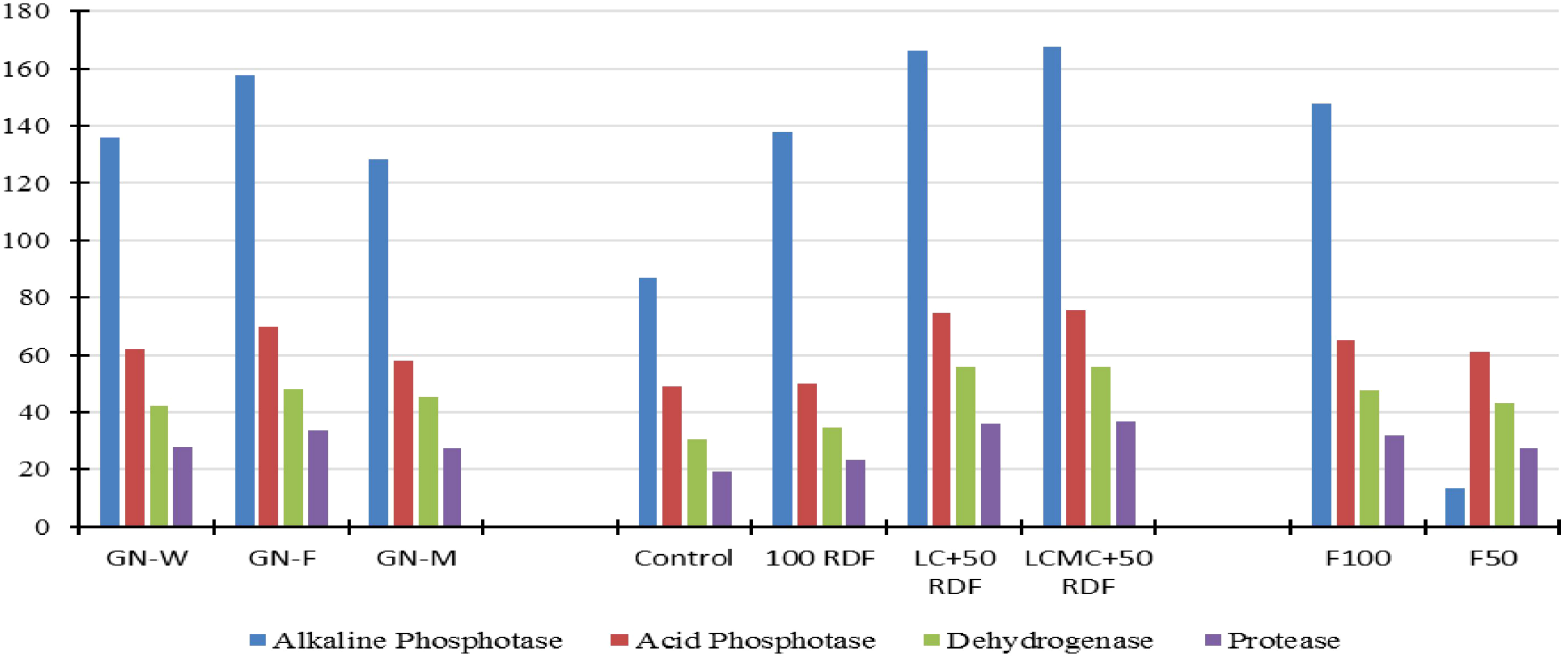
Effect of groundnut-based cropping systems (GBCSs) and system-mode bio-compost embedded nutrient management schedules (SBINMSs) on soil enzymes. Y-axis measuring units: alkaline phosphatase (µg *p*-nitrophenol·g^−1^ soil·h^−1^), acid phosphatase (µg *p*-nitrophenol·g^−1^ soil·h^−1^), dehydrogenase (µg TPF·g^−1^ soil·h^−1^), and protease (µg trypsin·g^−1^ soil·h^−1^).

Soil enzymatic activities, as shown in **Figure 7**, follow a similar trend of SMBC and SMBN. Plots with the GFCS had higher soil enzymatic activities than the GMCS and GWCS. Previous studies have also reported similar results where the cultivation of legumes increased the enzymatic activity of soil (Singh et al., 2008; Singh et al., 2021; Singh et al., 2022a). Soil enzymatic activity is positively correlated with microbial activity, as soil enzymes catalyze the biochemical reactions that are critical for microbial functions (DeLuca et al., 2019). In this study, organic manures along with chemical fertilizers remarkably improved the alkaline and acid phosphatase, dehydrogenase, and protease activities. The largest increase in soil enzymatic activities was observed in LCMC+RDF_50_ treatment, where leaf and cow dung-based compost was used as a nutrient source along with 50% RDF. The plots that received no fertilizers and manures (control) showed the lowest enzymatic activities. The F100 application to winter crops also increased soil enzymatic activities compared with F50. Hence, it is inferred that the integrated application of chemical fertilizers and organic manures, i.e., SBINMSs, is highly effective in enhancing the soil enzymatic activities (Bhupenchandra et al., 2022a; Bhupenchandra et al., 2022c).

### System productivity

**Figure 8:**
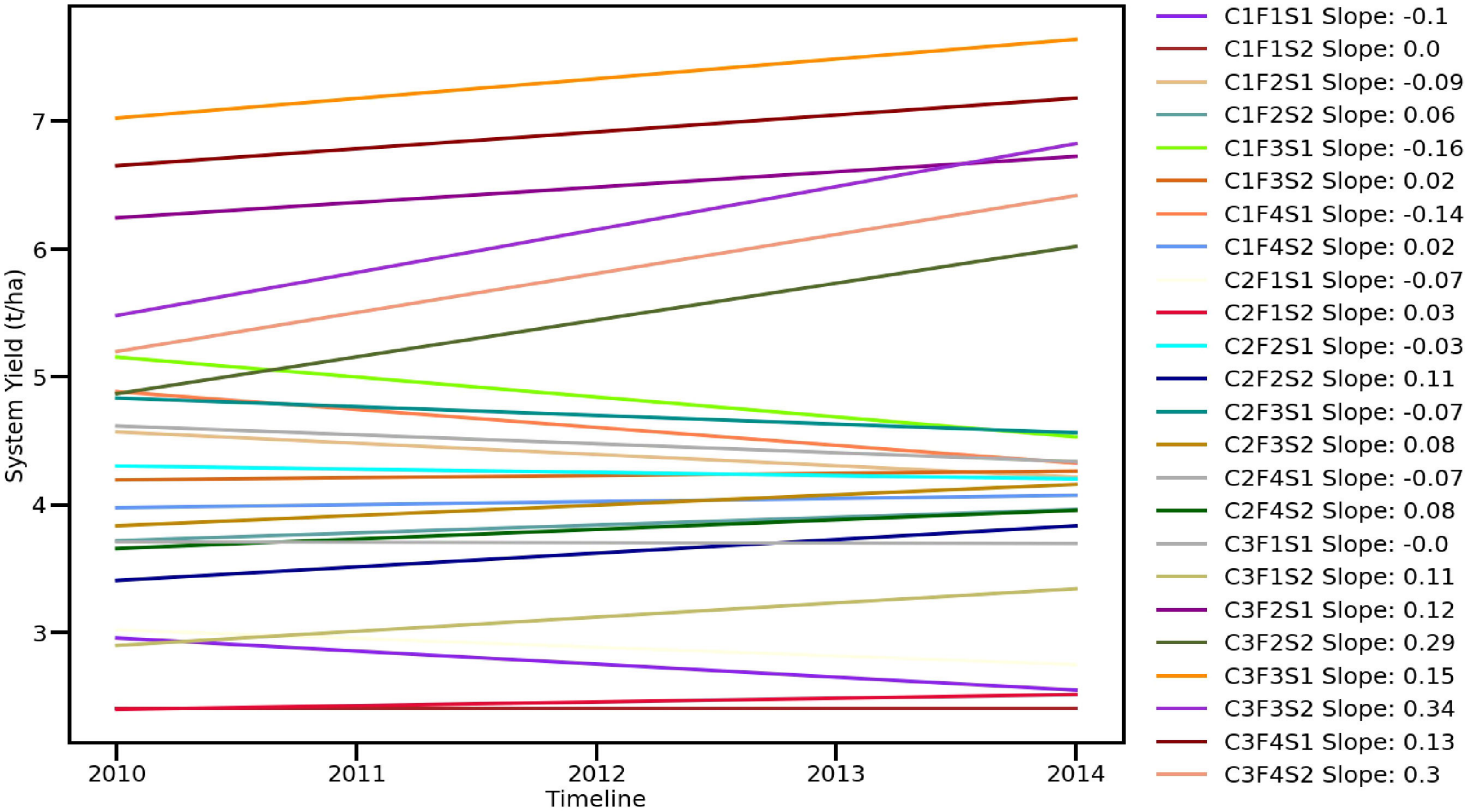
Effect of groundnut-based cropping systems (GBCSs) and system-mode bio-compost embedded nutrient management schedules (SBINMSs) on system productivity (t/ha).

**Figure 9:**
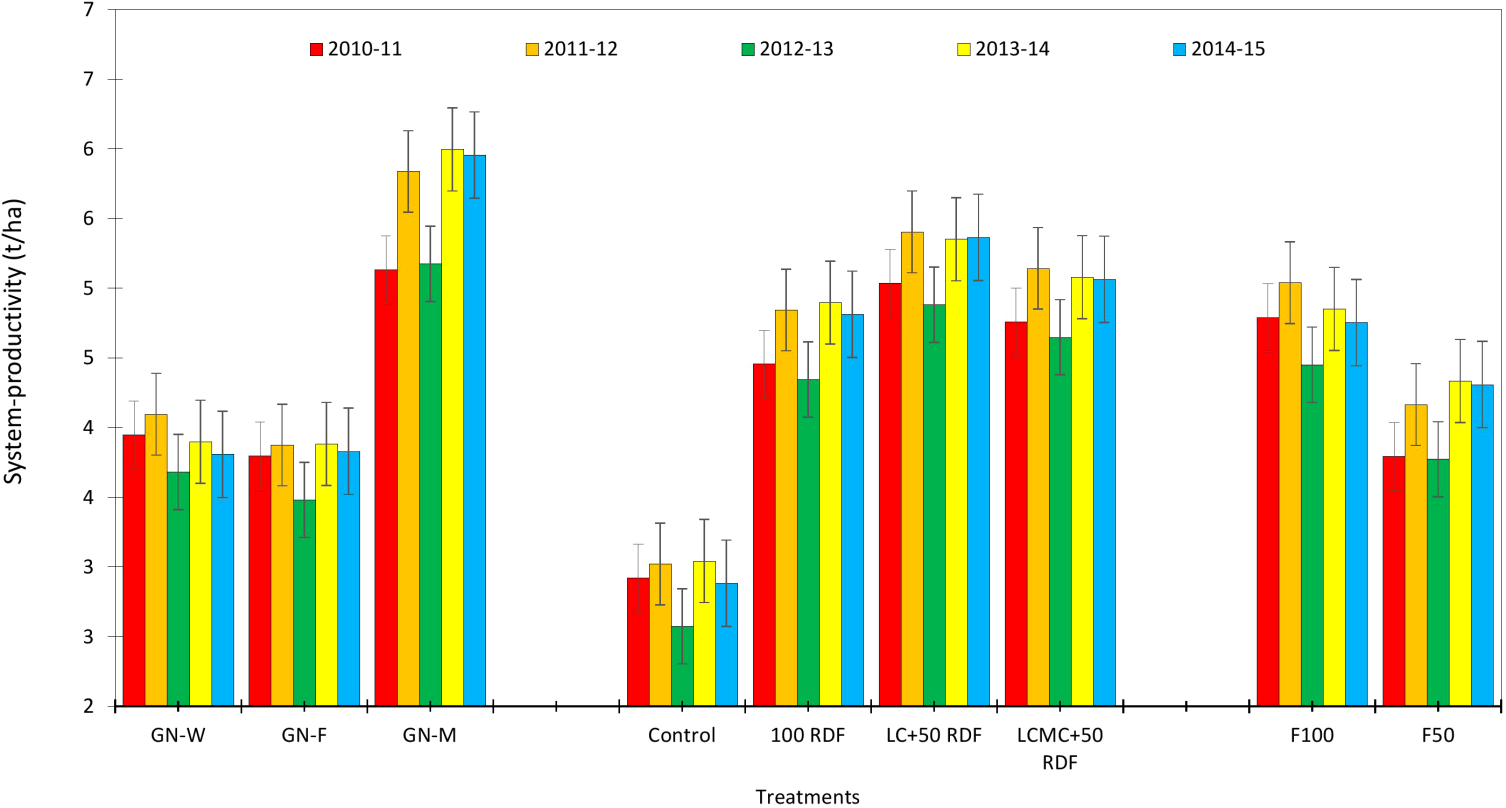
Effect of groundnut-based cropping systems (GBCSs) and system-mode bio-compost embedded nutrient management schedules (SBINMSs) on system productivity (GEY).

**Figure 10:**
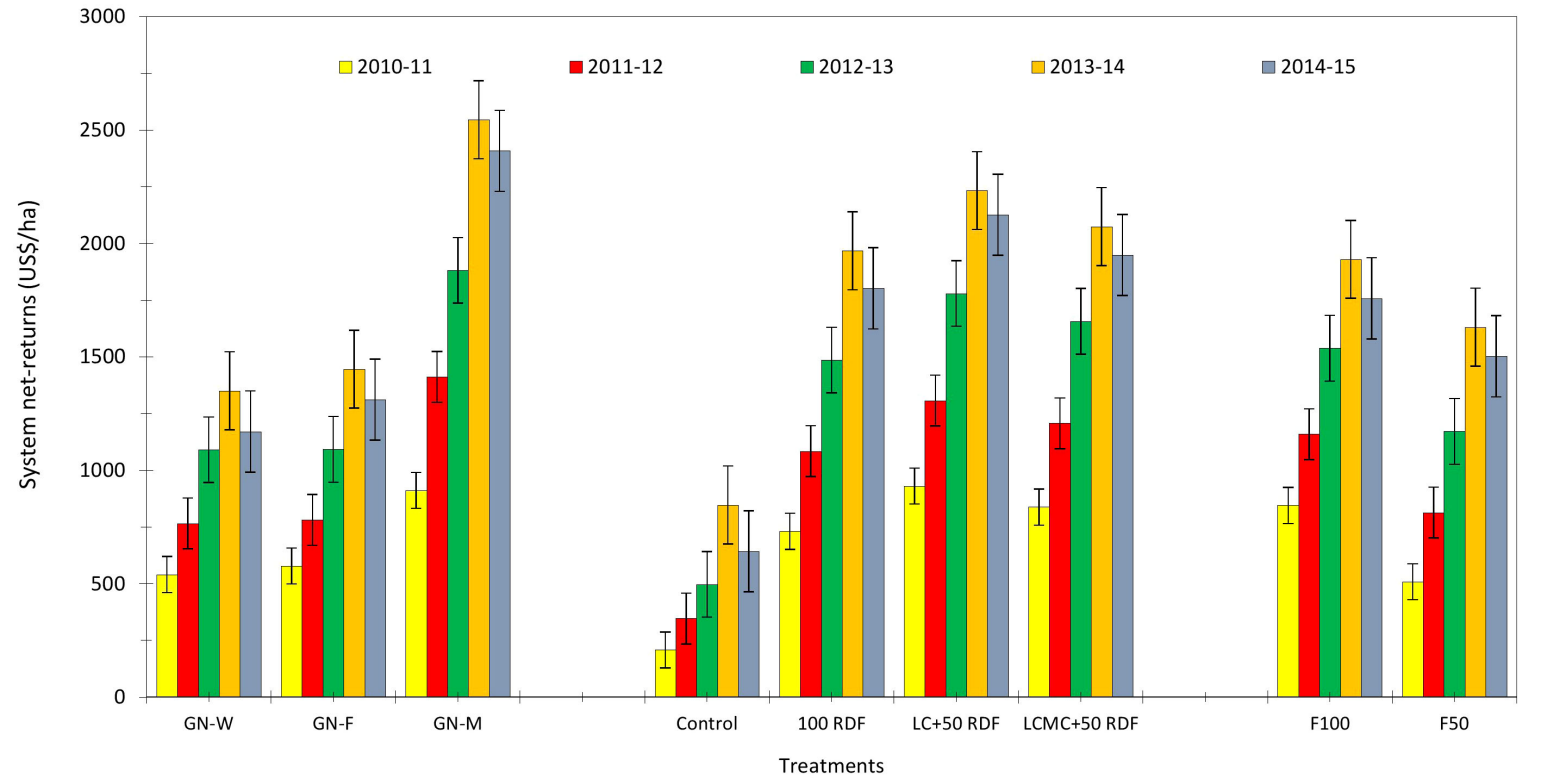
Effect of groundnut-based cropping systems (GBCSs) and system-mode bio-compost embedded nutrient management schedules (SBINMSs) on system net returns (US$/ha).

**Figure 11:**
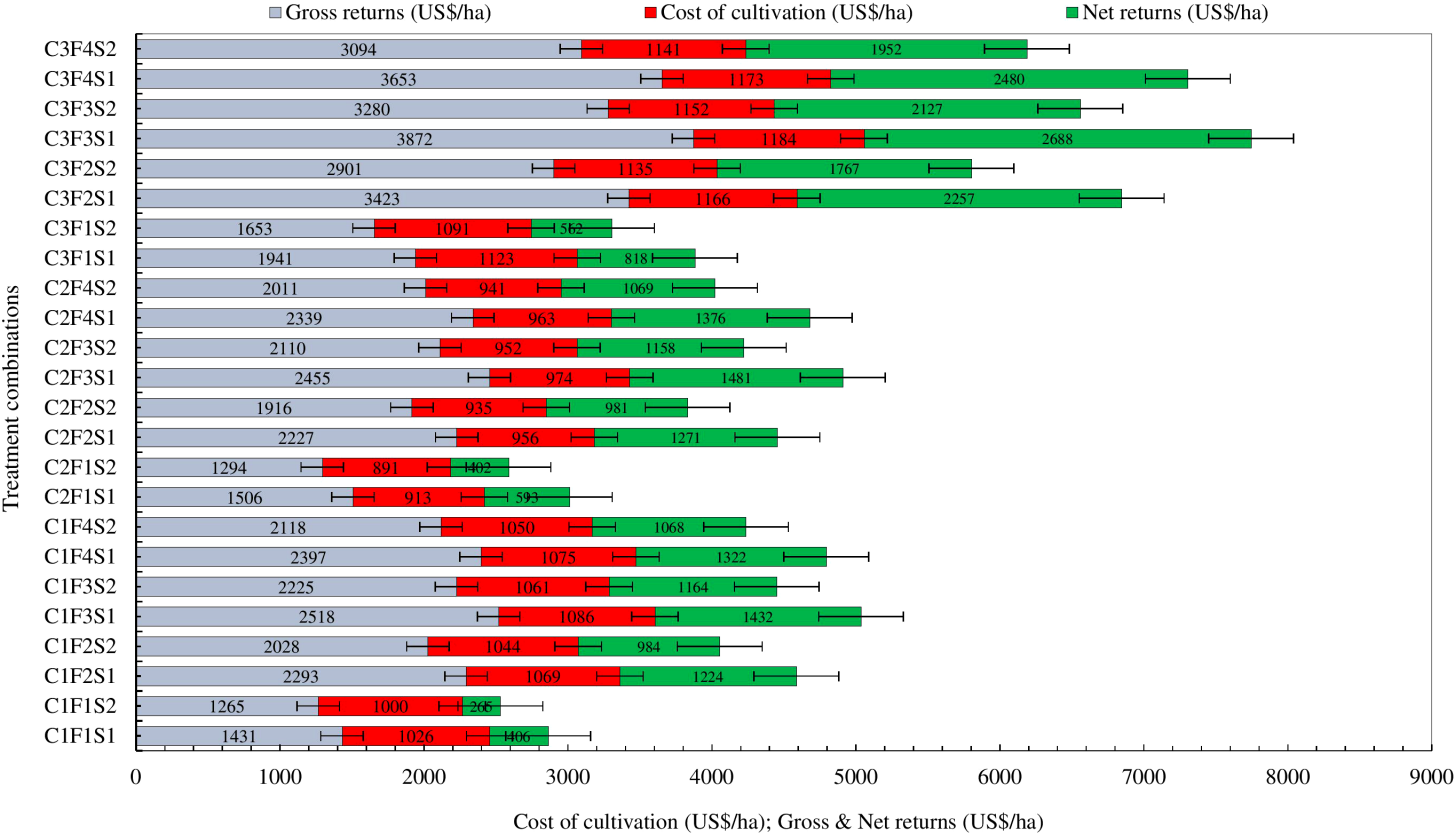
Effect of groundnut-based cropping systems (GBCSs) and system-mode bio-compost embedded nutrient management schedules (SBINMSs) on cost of cultivation (US$/ha) and gross and net returns (US$/ha) of various GBCSs (5-year av.).

**Figure 12:**
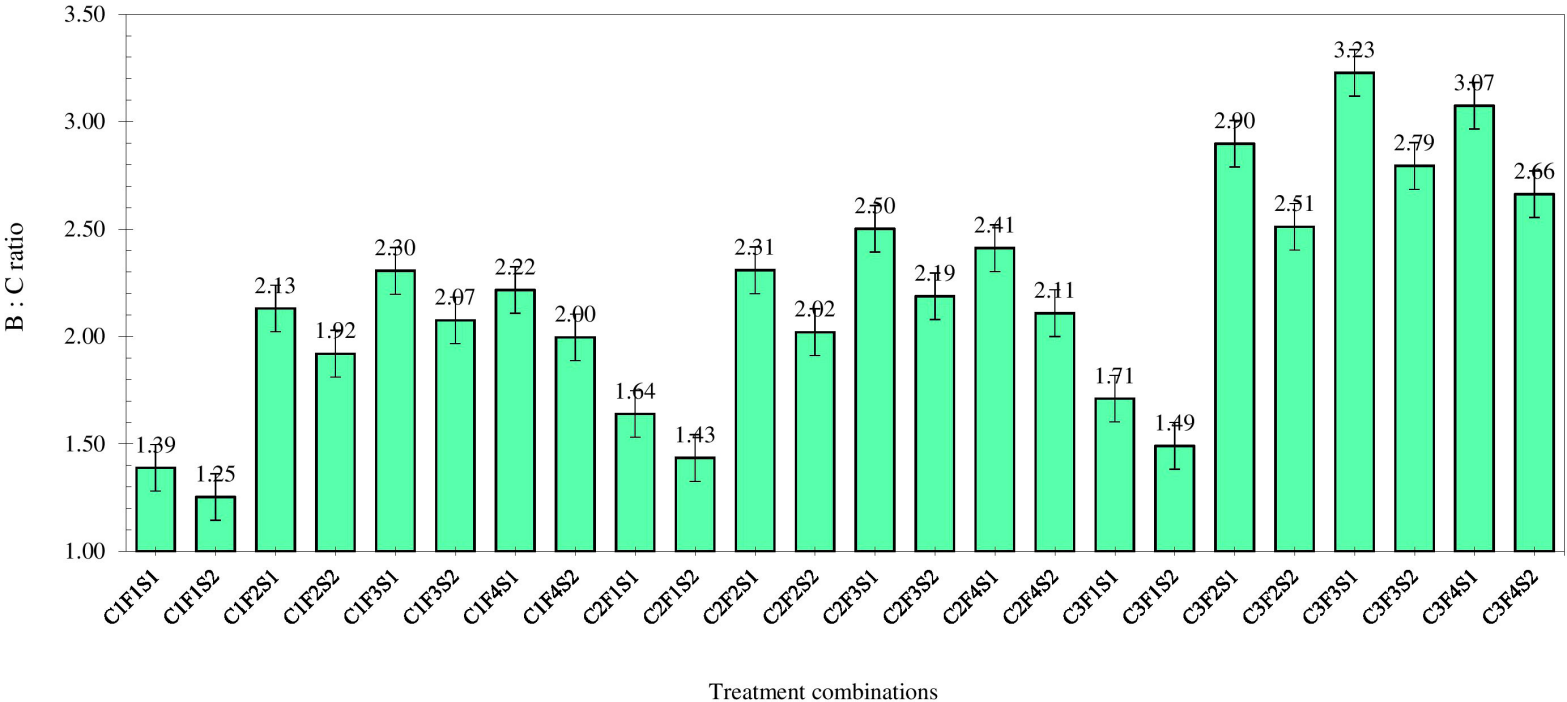
Effect of groundnut-based cropping systems (GBCSs) and system-mode bio-compost embedded nutrient management schedules (SBINMSs) on system B:C ratio (5-year av.).

Another major objective of this experiment was to study the effect of high-value crops’ embedded diversified cropping systems and different SBINMSs on system productivity. The overall effect of different treatments on system productivity is illustrated in **Figure 8**. Nine experimental treatments showed an overall decline in system productivity. It was lowest in 2012–2013. Among the three cropping systems, the GMCS recorded the highest system productivity (5.13–5.99 t/ha) in terms of GEY, which was 1.73 and 1.85 t/ha higher than that of the GWCS and GFCS, respectively (**Figure 9**). The market prices of the yield obtained from wheat, fenugreek, and marigold were taken into consideration to calculate the GEY. The GMCS also had higher system net returns over the years (**Figure 10**), with an average (5-year av.) system net returns of US$ 1,767–2,688/ha and system benefit:cost (B:C) ratio of 2.51–3.23 across different SBINMS practices despite of higher system cost of cultivation (US$ 1,135–1,184/ha) (**Figures 11**, **12**). Marigold received considerably higher prices in the market compared to wheat and fenugreek. Therefore, the system productivity of the GMCS was much higher (5.13–5.99 t/ha) compared to that of the GWCS (3.68–4.10 t/ha) and GFCS (3.48–3.88 t/ha) (**Figure 9**). On average, the highest system productivity, system net returns, and system B:C ratio were observed under C3F3S1 (GMCS with the application of LC at 5 t/ha + 50% RDF in groundnut and 100% RDF in marigold crop) followed by C3F4S1 (GMCS with the application of LCMC at 5 t/ha + 50% RDF in groundnut and 100% RDF in marigold crop) (**Figures 10**–**12**; **Supplementary Figure 1**). The SBINMSs, particularly with LC, were found superior compared with sole fertilizer use due to better nutrient supply and soil biological properties (Bhupenchandra et al., 2022c; Varatharajan et al., 2022). The plots treated with LC+RDF_50_ increased system productivity by ~80% and 12% compared with control and 100 RDF plots, respectively (**Figure 10**). Likewise, F100 also showed ~17% higher system productivity than F50. Among nutrient management practices, LC+RDF_50_ had the highest B:C ratio compared with LCMC+RDF_50_ (**Figure 12**). The highest B:C ratio was observed for treatments C3F3S1 and C3F4S2, whereas the lowest was observed for C1F1S2 and C1F1S1 (**Figure 12**). This can be attributed to the lower prices of LC compared to manure compost.

### Which-won-where

**Figure 13:**
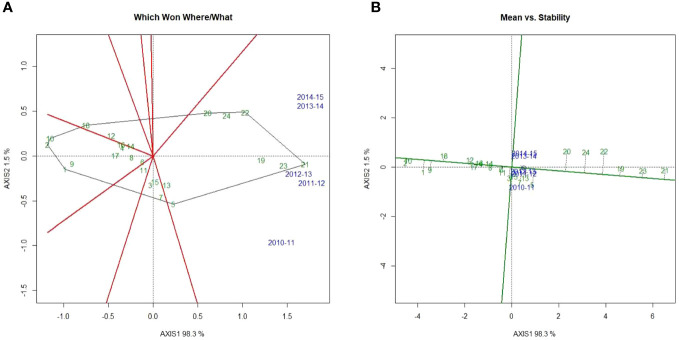
GGE biplots. **(A)** Which-won-where polygon displays superior treatments, and **(B)** mean *vs.* stability biplot displays the performance of the treatment and its stability. X axis: principal component 1—system yield. Y axis: principal component 2—gross returns.

To analyze the system productivity, different types of GGE biplots were generated, the results of which are mentioned here (Gabriel, 1971; Yan and Tinker, 2006). The “which-won-where/what” polygon was employed to identify the best treatment for each environment (year) (**Figure 13A**). Almost all of the variations among different treatments related to system productivity were explained by only one factor, system yield (98.3%), and gross returns explained a further 1.5%. In terms of system yield and net returns, the GMCS significantly outperformed the GWCS and GFCS throughout the 5 years. Treatment LC+50 for the wet season and treatment F100 for the winter season were observed to be the most superior treatments for all the years. The polygon clustered the treatments in five different clusters, which are given in the order of performance (“a” performed the poorest and “e” performed the best): a) treatments 1, 2, 9, 10, 18, and 17 (all controls); b) treatments 4, 6, 8, 12, 14, and 16 (GFCS system with F50 in winter); c) treatments 3, 5, 7, 11, 13, and 15 (GWCS with F100 in winter); d) treatments 20, 22, and 24 (GMCS with F50 in winter); and e) treatments 19, 21, and 23 (GMCS with F100 in winter). These results suggested that the application of 100F in the winter season plays an important role in increasing the system-productivity yield and system net returns. Overall, it can be suggested that by adopting the SBINMS approach embedded with leaf compost, the annual use of inorganic fertilizers can be reduced by ~25% in the system mode, and on top of that, system productivity can also be enhanced appropriately. Hence, the selection of an appropriate cropping system adds to the benefit of higher system productivity.

### Mean *vs.* stability

The mean *vs.* stability biplot was used to identify the performance stability of the treatments for the tested environments. Treatments were ranked along the average-tester axis (ATA). The circle indicates the average treatment. Based on the mean performance of system productivity and system net returns, the treatments above average were ranked as 21>23>19>22>24>20>5>13>7 (**Figure 13B**). All other treatments gave below-average system productivity and system net returns. The highest yield stability and profitability were recorded for treatments 21, 23, and 19 in the years 2013–2014 and 2014–2015. For the years 2010–2011, 2011–2012, and 2012–2013, the highest yield stability and profitability were recorded for treatments 5, 13, and 7. The treatment stability is reciprocally related to the length of the projection (regardless of direction) away from ATA (Bana et al., 2022). Treatments 21, 23, and 19 were more stable compared to 22, 24, and 20.

### Ranking treatments

The ideal treatment for system productivity and system net returns was identified through the genotype ranking biplot (**Supplementary Figure 2A**). The treatment closest to the concentric ring is considered the best treatment (Bana et al., 2020). For system productivity, all the treatments were located in the same circle; therefore, treatments positioned closest to the arrowhead were considered the most ideal treatments. The ranking for the top 10 ideal treatments for system productivity was 21>23>19>24>22>20>15>3>13>7. Likewise, the raking for the ideal treatment for system net returns was 21>23>19>22>24>20>13>5>7>11>3>14>6>16>8>12>4>17>18> 9>10>1>2.

### Ranking environments

The most ideal environment was the one that was closest to the co-centric sphere, as it scored the highest rank (**Supplementary Figure 2B**). For system productivity, the ranking of environments, i.e., years, was 2011–2012>2012–2013>2013–2014>2014–2015>2010–2011. For system net returns, the ranking of environments, i.e., years, was 2013–2014>2014–2015>2012–2013>2011–2012>2010–2011.

## Conclusions

The current study aimed at pinpointing the most appropriate high-value crops’ embedded GBCS and system-mode INM schedules for achieving higher system productivity while preserving soil bio-fertility in semi-arid South Asia. It was suggested that the SBINMS approach of leaf compost (5 t/ha) along with 50% RDF in the GMCS had a significantly positive correlation with system productivity and soil biological and chemical health. The use of leaf compost in system mode (SBINMSs) proved a more cost-effective and efficient alternative than manure compost, which may reduce the annual use of inorganic fertilizers by ~25% while using LC+50% RDF in groundnut and 100% RDF in winter-season crops. The LC+50% RDF also enhanced the system productivity and soil biological and chemical properties. Overall, the high-value crop (marigold) embedded GMCS along with LC+50% RDF in wet-season crops and 100% RDF in winter-season crops proved more productive with system-productivity of 5.13–5.99 t/ha and remunerative with system net returns of US$ 1,767–2,688/ha in addition to improved soil bio-fertility indicators in the semi-arid climate of South Asia. Understanding the effect of diversified production systems and innovative nutrient management schedules on environmental footprints (greenhouse gas emissions), water productivity, energy use efficiency, and carbon footprints would be an important future research area based on the work of the present study. Furthermore, calibration and validation of cropping system models from the present study to identify ideal future management of GBCS under changing climate and resource-availability scenarios, and diverse representative concentration pathways, would be other future research areas that the present work could contribute to.

## Data availability statement

The original contributions presented in this study are included in the article/**Supplementary Material**. Further inquiries can be directed to the corresponding author.

## Author contributions

RB: Conceptualization, Data curation, Formal analysis, Investigation, Methodology, Project administration, Resources, Supervision, Writing – original draft, Writing – review & editing. AC: Conceptualization, Data curation, Formal analysis, Investigation, Methodology, Project administration, Supervision, Validation, Writing – original draft, Writing – review & editing. RN: Conceptualization, Investigation, Methodology, Resources, Writing – original draft. BK: Conceptualization, Investigation, Methodology, Writing – original draft. SS: Data curation, Formal analysis, Investigation, Methodology, Writing – original draft. SG: Data curation, Formal analysis, Investigation, Methodology, Writing – original draft. RB: Conceptualization, Methodology, Writing – original draft. DS: Data curation, Formal analysis, Investigation, Writing – original draft. DR: Conceptualization, Methodology, Project administration, Resources, Writing – original draft.

## References

[B1] Alkorta I. Aizpurua A. Riga P. Albizu I. Amézaga I. Garbisu C. (2003). Soil enzyme activities as biological indicators of soil health. Rev. Env. Health 18, 65–73. doi: 10.1515/REVEH.2003.18.1.65 12875512

[B2] Ambast S. K. Tyagi N. K. Raul S. K. (2006). Management of declining groundwater in the Trans Indo-Gangetic Plain (India): Some options. Agric. Water Manage. 82, 279–296. doi: 10.1016/j.agwat.2005.06.005

[B3] APEDA (2023) Agricultural and Processed Food Products Export Development Authority (APEDA) (New Delhi: Ministry of Commerce and Industry, Govt. of India). Available at: http://apeda.gov.in (Accessed May 1, 2023).

[B4] Balota E. L. Colozzi-Filho A. Andrade D. S. Dick R. P. (2003). Microbial biomass in soils under different tillage and crop rotation systems. Biol. Fertil. Soils. 38, 15–20. doi: 10.1007/s00374-003-0590-9

[B5] Bana R. S. Faiz M. A. Sangwan S. Choudhary A. K. Bamboriya S. D. Godara S.. (2023). Triple-zero tillage and system intensification lead to enhanced productivity, micronutrient biofortification and moisture-stress tolerance ability in chickpea in a pearlmillet-chickpea cropping system of semi-arid climate. Scientific Reports 13, 10226. doi: 10.1038/s41598-023-36044-0 37353506 PMC10290053

[B6] Bana R. S. Gautam R. C. Rana K. S. (2012). Effect of different organic sources on productivity and quality of pearl millet and their residual effect on wheat. Ann. Agric. Sci. 33, 126–130.

[B7] Bana R. S. Jat G. S. Grover M. Bamboriya S. D. Singh. D. Bansal R.. (2022). Foliar nutrient supplementation with micronutrient-embedded fertilizer increases biofortification, soil biological activity and productivity of eggplant. Sci. Rep. 12, 5146. doi: 10.1038/s41598-022-09247-0 35338233 PMC8956703

[B8] Bana R. S. Pooniya V. Choudhary A. K. Rana K. S. Tyagi V. K. (2016). Influence of organic nutrient sources and moisture management on productivity, biofortification and soil health in pearl millet (*Pennisetum glaucum*) + clusterbean (*Cyamopsis tetragonaloba*) intercropping system of semi-arid India. Indian J. Agric. Sci. 86, 1418–1425. doi: 10.56093/ijas.v86i11.62895

[B9] Bana R. S. Sepat S. Rana K. S. Pooniya V. Choudhary A. K. (2018). Moisture-stress management under limited and assured irrigation regimes in wheat (*Triticum aestivum*): Effects on crop productivity, water use efficiency, grain quality, nutrient acquisition and soil fertility. Indian J. Agric. Sci. 88, 1606–1612. doi: 10.56093/ijas.v88i10.84237

[B10] Bana R. S. Shivay Y. S. Tyagi V. K. (2015). Effect of summer forage crops and phosphogypsum–enriched urea on soil quality, nitrogen-use efficiency and quality of *Basmati* rice (*Oryza sativa*) and their residual effect on succeeding wheat (*Triticum aestivum)* . Indian J. Agric. Sci. 85, 531–538. doi: 10.56093/ijas.v85i4.47934

[B11] Bana R. S. Singh D. Nain M. S. Kumar H. Kumar V. Sepat S. (2020). Weed control and rice yield stability studies across diverse tillage and crop establishment systems under on-farm environments. Soil Till. Res. 204, 104729. doi: 10.1016/j.still.2020.104729

[B12] Bhupenchandra I. Basumatary A. Choudhary A. K. Kumar A. Sarkar D. Chongtham S. K.. (2022a). Elucidating the impact of boron fertilization on soil physico-chemical and biological entities under cauliflower-cowpea-okra cropping system in an eastern Himalayan acidic Inceptisol. Front. Microbiol. 13. doi: 10.3389/fmicb.2022.996220 PMC9676249 36419419

[B13] Bhupenchandra I. Chongtham S. K. Basumatary A. Singh A. H. Das A. Choudhary A. K.. (2022b). Changes in soil properties, productivity and profitability as influenced by the adoption of site-specific integrated crop management technology in turmeric (*Curcuma longa* L.) in eastern Himalayan acidic Inceptisol. Indust. Crops Prod. 180, 114745. doi: 10.1016/j.indcrop.2022.114745

[B14] Bhupenchandra I. Chongtham S. K. Devi E. L. Ramesh R. Choudhary A. K. Devi S. M.. (2022c). Role of biostimulants in mitigating the effects of climate change on crop performance. Front. Plant Sci. 13. doi: 10.3389/fpls.2022.967665 PMC9634556 36340395

[B15] Borase D. N. Nath C. P. Hazra K. K. Senthilkumar M. Singh S. S. Praharaj C. S.. (2020). Long-term impact of diversified crop rotations and nutrient management practices on soil microbial functions and soil enzymes activity. Ecol. Indic. 114, 106322. doi: 10.1016/j.ecolind.2020.106322

[B16] Bouyoucos C. J. (1962). Hydrometer method improved for making particle size analysis of soil. Agron. J. 54, 464–465. doi: 10.1016/j.ecolind.2020.106322

[B17] Brookes P. C. Landman A. Pruden G. Jenkinson D. S. (1985). Chloroform fumigation and the release of soil nitrogen: a rapid direct extraction method to measure microbial biomass nitrogen in soil. Soil Biol. Biochem. 17, 837–842. doi: 10.1016/0038-0717(85)90144-0

[B18] Casida L. E. Klein D. A. Santoro T. (1964). Soil dehydrogenase activity. Soil Sci. 98, 371–376. doi: 10.1097/00010694-196412000-00004

[B19] Chinnadurai C. Gopalaswamy G. Balachandar D. (2014). Long-term effects of nutrient management regimes on abundance of bacterial genes and soil biochemical processes for fertility sustainability in a semi-arid tropical alfisol. Geoderma 232, 563–572. doi: 10.1016/j.geoderma.2014.06.015

[B20] Choudhary A. K. Rahi S. (2018). Organic cultivation of high yielding turmeric (*Curcuma longa* L.) cultivars: A viable alternative to enhance rhizome productivity, profitability, quality and resource-use efficiency in monkey-menace areas of north-western Himalayas. Indust. Crops Prod. 124, 495–504. doi: 10.1016/j.indcrop.2018.07.069

[B21] Choudhary A. K. Varatharajan T. Rohullah Bana R. S. Pooniya V. Dass A.. (2020). Integrated crop management technology for enhanced productivity, resource-use efficiency and soil health in legumes – A review. Indian J. Agric. Sci. 90 (10), 1839–1849. doi: 10.56093/ijas.v90i10.107882

[B22] Das T. K. Bhattacharyya R. Sharma A. R. Das S. Saad A. A. Pathak H. (2013). Impacts of conservation agriculture on total soil organic carbon retention potential under an irrigated agro-ecosystem of the western Indo-Gangetic Plains. Eur. J. Agron. 51, 34–42. doi: 10.1016/j.eja.2013.07.003

[B23] DeLuca T. H. Pingree M. R. A. Gao S. (2019). “Assessing soil biological health in forest soils,” in Developments in Soil Science, vol. 36 . Ed. Busse M. Giardina C. P. Morris D. M. Dumroese D. S. (Elsevier), 397–426. https://www.sciencedirect.com/science/article/abs/pii/B9780444639981000161?via%3Dihub

[B24] Doran J. W. (2002). Soil health and global sustainability: translating science into practice. Agric. Ecosyst. Environ. 88, 119–127. doi: 10.1016/S0167-8809(01)00246-8

[B25] Doran J. W. Zeiss M. R. (2000). Soil health and sustainability: managing the biotic component of soil quality. Appl. Soil Ecol. 15, 3–11. doi: 10.1016/S0929-1393(00)00067-6

[B26] FAOSTAT (2023) Food and agricultural organization statistics database (Faostat). Available at: http://faostat.fao.org (Accessed May 1, 2023).

[B27] Gabriel K. R. (1971). The biplot graphic display of matrices with application to principal component analysis. Biometrika 58, 453–467. doi: 10.1093/biomet/58.3.453

[B28] Ghosh P. K. Hazra K. K. Venkatesh M. S. Nath C. P. Singh J. Nadarajan N. (2017). Increasing soil organic carbon through crop diversification in cereal–cereal rotations of indo-gangetic plain. Proc. Nat. Acad. Sci. India Sec. B Biol. 89, 429–440. doi: 10.1007/s40011-017-0953-x

[B29] Gupta G. Dhar S. Kumar A. Choudhary A. K. Dass A. Sharma V. K.. (2022). Microbes-mediated integrated nutrient management for improved rhizo-modulation, pigeonpea productivity, and soil bio-fertility in a semi-arid agro-ecology. Front. Microbiol. 13. doi: 10.3389/fmicb.2022.924407 PMC9520524 36187978

[B30] Harish M. N. Choudhary A. K. Bhupenchandra I. Dass A. Rajanna G. A. Singh V. K.. (2022a). Double zero-tillage and foliar-P nutrition coupled with bio-inoculants enhance physiological photosynthetic characteristics and resilience to nutritional and environmental stresses in maize–wheat rotation. Front. Plant Sci. 13. doi: 10.3389/fpls.2022.959541 PMC9520575 36186084

[B31] Harish M. N. Choudhary A. K. Kumar S. Dass A. Singh V. K. Sharma V. K.. (2022b). Double zero-tillage and foliar-P fertilization coupled with microbial-inoculants lead to improved maize productivity and quality in a maize–wheat. Scientific Reports 12, 3161. doi: 10.1038/s41598-022-07148-w 35210519 PMC8873388

[B32] Heba M. N. Rana D. S. Choudhary A. K. Dass A. Rajanna G. A. Pande. P. (2021). Influence of sulphur and zinc nutrition on productivity, quality and biofortification in groundnut (*Arachis hypogea* L.) in south-Asian Alluvial soil. J. Plant Nutr. 44 (8), 1151–1174. doi: 10.1080/01904167.2020.1849289

[B33] Heba M. N. Rana D. S. Choudhary A. K. Rajpoot S. K. Paul T. (2016). Sulphur and Zn management in groundnut (*Arachis hypogaea*)–wheat (*Triticum aestivum*) cropping system: Direct effects on system productivity and residual effects on yield, energetics and Zn biofortification in wheat. Indian J. Agric. Sci. 86 (4), 441–447.

[B34] IOPEP (2017). Indian Oilseeds and Produce Export Promotion Council (Indian Oilseeds and Produce Export Promotion Council, Ministry of Commerce, Govt. of India, New Delhi) 30. Available at: https://iopepc.org/products-edible-oils.php

[B35] Jackson M. L. (1958). Soil Chemical Analysis (New Jercey: Prentice Hall), 498.

[B36] Jackson M. L. (1958). Soil Chemical Analysis (New Delhi: Prentice Hall of India Private Limited), 187.

[B37] Jenkinson D. S. Powlson D. S. (1976). The effects of biocidal treatments on metabolism in soil—I. Fumigation with chloroform. Soil Biol. Biochem. 8, 167–177. doi: 10.1016/0038-0717(76)90001-8

[B38] Karlen D. L. Mausbach M. J. Doran J. W. Cline R. G. Harris R. F. Schuman G. E. (1997). Soil quality: A concept, definition, and framework for evaluation (a guest editorial). Soil Sci. Soc Am. J. 61, 4–10. doi: 10.2136/sssaj1997.03615995006100010001x

[B39] Kumar A. Rana K. S. Choudhary A. K. Bana R. S. Sharma V. K. Gupta G.. (2022). Sole- or dual-crop basis residue-mulching and Zn-fertilization lead to improved productivity, rhizo-modulation and soil health in zero-tilled pigeonpea-wheat cropping system. J. Soil Sci. Plant Nutr. 22 (2), 1193–1214. doi: 10.1007/s42729-021-00723-6

[B40] Kumar A. Rana K. S. Choudhary A. K. Bana R. S. Sharma V. K. Prasad S.. (2021). Energy budgeting and carbon footprints of zero-tilled pigeonpea–wheat cropping system under sole or dual crop basis residue mulching and Zn-fertilization in a semi-arid agro-ecology. Energy 231, 120862. doi: 10.1016/j.energy.2021.120862

[B41] Kumar U. Shahid M. Tripathi R. Mohanty S. Kumar A. Bhattacharyya R.. (2017). Variation of functional diversity of soil microbial community in sub-humid tropical rice-rice cropping system under long-term organic and inorganic fertilization. Ecol. Indic. 73, 536–543. doi: 10.1016/j.ecolind.2016.10.014

[B42] Ladd J. N. Butler J. H. A. (1972). Short-term assays of soil proteolytic enzyme activities using proteins and dipeptide derivatives as substrates. Soil Biol. Biochem. 4, 19–30. doi: 10.1016/0038-0717(72)90038-7

[B43] Lazcano C. Gómez-Brandón M. Revilla P. Domínguez J. (2013). Short-term effects of organic and inorganic fertilizers on soil microbial community structure and function. Biol. Fertil. Soils. 49, 723–733. doi: 10.1007/s00374-012-0761-7

[B44] Lindsay W. L. Norvell W. A. (1978). Development of a dtpa soil test for zinc, iron, manganese, and copper. Soil Sci. Soc Am. J. 42, 421–428. doi: 10.2136/sssaj1978.03615995004200030009x

[B45] Liu Y. Wu L. Baddeley J. A. Watson C. A. (2011). Models of biological nitrogen fixation of legumes. Agron. Sust. Dev. 31, 155–172. doi: 10.1051/agro/2010008

[B46] Liu E. Yan C. Mei X. He W. Bing S. H. Ding L.. (2010). Long-term effect of chemical fertilizer, straw, and manure on soil chemical and biological properties in northwest China. Geoderma 158, 173–180. doi: 10.1016/j.geoderma.2010.04.029

[B47] Mauchline T. H. Chedom-Fotso D. Chandra G. Samuels T. Greenaway N. Backhaus A.. (2015). Pseudomonas genome diversity in wheat rhizospheres. Environ. Microbiol. 17, 4764–4778. doi: 10.1111/1462-2920.13038 26337499 PMC4832304

[B48] Mayer J. Buegger F. Jensen E. S. Schloter M. Heß J. (2004). Turnover of grain legume N rhizodeposits and effect of rhizodeposition on the turnover of crop residues. Biol. Fertil. Soils. 39, 153–164. doi: 10.1007/s00374-003-0694-2

[B49] Naragund R. Singh Y. V. Bana R. S. Choudhary A. K. Jaiswal P. (2020). Influence of crop establishment and microbial inoculants on profitability and nutrient concentrations of summer greengram (*Vigna radiata*). Indian J. Agric. Sci. 90, 1348–1351. doi: 10.56093/ijas.v90i7.105618

[B50] Nath C. P. Das T. K. Bhattacharyya R. Pathak H. Paul S. Chakraborty D.. (2017). Nitrogen effects on productivity and soil properties in conventional and zero tilled wheat with different residue management. Proc. Natl. Acad. Sci. India Sec. B Biol. Sci. 89, 123–135.

[B51] Nath C. P. Hazra K. K. Kumar N. Praharaj C. S. Singh S. S. Singh U.. (2019). Including grain legume in rice–wheat cropping system improves soil organic carbon pools over time. Ecol. Engg. 129, 144–153. doi: 10.1016/j.ecoleng.2019.02.004

[B52] Noman H. M. Rana D. S. Choudhary A. K. Rajpoot S. Paul T. (2016). Sulphur and Zn management in groundnut (Arachis hypogaea)–wheat (Triticum aestivum) cropping system: direct effects on system productivity and residual effects on yield, energetics and Zn biofortification in wheat. Indian J. Agric. Sci. 86, 441–447. doi: 10.56093/ijas.v86i4.57434

[B53] Olsen S. R. Cole C. V. Watanabe F. S. Dean L. A. (1954). Estimation of available phosphorus in soil by extraction with sodium bicarbonate (Washington: USDA, Washington, USA), 72–75. USDA Circular No. 939. USDA.

[B54] Perelo L. W. Munch J. C. (2005). Microbial immobilisation and turnover of ^13^C labelled substrates in two arable soils under field and laboratory conditions. Soil Biol. Biochem. 37, 2263–2272. doi: 10.1016/j.soilbio.2005.02.039

[B55] Rajanna G. A. Dass A. Singh V. K. Choudhary A. K. Venkatesh P. Babu S.. (2023). Energy and carbon budgeting in a soybean–wheat system in different tillage, irrigation and fertilizer management practices in south-Asian semi-arid agroecology. Eur. J. Agron. 148, 126877. doi: 10.1016/j.eja.2023.126877

[B56] Rajpoot S. K. Rana D. S. Choudhary A. K. (2021). Crop and water productivity, energy auditing, carbon footprints and soil health indicators of *Bt*-cotton transplanting led system intensification. J. Environ. Manage. 300, 113732. doi: 10.1016/j.jenvman.2021.113732 34537560

[B57] Rana K. S. Choudhary A. K. Sepat S. Bana R. S. Dass A. (2014). Methodological and Analytical Agronomy (New Delhi: IARI), 276.

[B58] Ren F. Sun N. Xu M. Zhang X. Wu L. Xu M. (2019). Changes in soil microbial biomass with manure application in cropping systems: a meta-analysis. Soil Till. Res. 194, 104291. doi: 10.1016/j.still.2019.06.008

[B59] Richardville K. Egel D. Flachs A. Jaiswal A. Perkins D. Thompson A.. (2022). Closing the loop in the city: Leaf mold compost reduces waste, improves soil and microbial properties, and increases tomato productivity. Urban Agric. Regional Food Syst. 7, e220022.

[B60] Saha R. Ghosh P. K. (2013). Soil organic carbon stock, moisture availability and crop yield as influenced by residue management and tillage practices in maize–mustard cropping system under hill agro-ecosystem. Natl. Acad. Sci. Lett. 36, 461–468. doi: 10.1007/s40009-013-0158-7

[B61] Selim M. M. (2020). Introduction to the integrated nutrient management strategies and their contribution to yield and soil properties. Int. J. Agron. 2020, 1–14. doi: 10.1155/2020/2821678

[B62] Sharma S. Padbhushan R. Kumar U. (2019). Integrated nutrient management in rice–wheat cropping system: an evidence on sustainability in the Indian subcontinent through meta-analysis. Agronomy 9, 71. doi: 10.3390/agronomy9020071

[B63] Sharma N. Singhvi R. (2017). Effects of chemical fertilizers and pesticides on human health and environment: A review. Int. J. Agric. Environ. Biotechnol. 10, 675–680. doi: 10.5958/2230-732X.2017.00083.3

[B64] Singh U. Choudhary A. K. Sharma S. (2020). Comparative performance of conservation agriculture vis-a-vis organic and conventional farming in enhancing plant attributes and rhizospheric bacterial diversity in *Cajanus cajan*: A field study. Eur. J. Soil Biol. 99, 103197. doi: 10.1016/j.ejsobi.2020.103197

[B65] Singh U. Choudhary A. K. Sharma S. (2021). Agricultural practices modulate the bacterial communities, and nitrogen cycling bacterial guild in rhizosphere: Field experiment with soybean. J. Sci. Food Agric. 101 (7), 2687–2695. doi: 10.1002/jsfa.10893 33070344

[B66] Singh U. Choudhary A. K. Sharma S. (2022a). A 3-year field study reveals that agri-management practices drive the dynamics of dominant bacterial taxa in the rhizosphere of *Cajanus cajan* . Symbiosis 86 (2), 215–227. doi: 10.1007/s13199-022-00834-3

[B67] Singh U. Choudhary A. K. Varatharajan T. Sharma S. (2022b). Agri-management practices affect the abundance of markers of phosphorus cycle in soil: Case study with pigeonpea and soybean. J. Soil Sci. Plant Nutr. 22, 3012–3020. doi: 10.1007/s42729-022-00863-3.2022

[B68] Singh B. Kaur R. Singh K. (2008). Characterization of *Rhizobium* strain isolated from the roots of *Trigonella foenumgraecum* (fenugreek). Afr. J. Biotechnol. 7, 3671–3676.

[B69] Six J. Frey S. D. Thiet R. K. Batten K. M. (2006). Bacterial and fungal contributions to carbon sequestration in agroecosystems. Soil Sci. So. Am. J. 70, 555–569. doi: 10.2136/sssaj2004.0347

[B70] Sreedevi P. D. Sreekanth P. D. Khan H. H. Ahmed S. (2013). Drainage morphometry and its influence on hydrology in an semi arid region: Using SRTM data and GIS. Environ. Earth Sci. 70, 839–848. doi: 10.1007/s12665-012-2172-3

[B71] Srinivasarao C. Singh S. P. Kundu S. Abrol V. Lal R. Abhilash P. C.. (2021). Integrated nutrient management improves soil organic matter and agronomic sustainability of semiarid rainfed Inceptisols of the Indo-Gangetic plains. J. Plant Nutr. Soil Sci. 184, 562–572. doi: 10.1002/jpln.202000312

[B72] Srinivasarao C. Venkateswarlu B. Lal R. Singh A. K. Kundu S. (2013). Sustainable management of soils of dryland ecosystems of India for enhancing agronomic productivity and sequestering carbon. Adv. Agron. 121, 253–325. doi: 10.1016/B978-0-12-407685-3.00005-0

[B73] Tabatabai M. A. Bremner J. M. (1969). Use of p-nitrophenyl phosphate for assay of soil phosphatase activity. Soil Biol. Biochem. 1, 301–307. doi: 10.1016/0038-0717(69)90012-1

[B74] Tamilselvi S. M. Chinnadurai C. Ilamurugu K. Arulmozhiselvan K. Balachandar D. (2017). Effect of long-term nutrient management on biological and biochemical properties of semi-arid tropical Alfisol during maize crop developmental stages. Ecol. Indic 48, 76–87. doi: 10.1016/j.ecolind.2014.08.001

[B75] Van Bruggen A. H. Semenov A. M. (2000). In search of biological indicators for soil health and disease suppression. Appl. Soil Ecol. 15, 13–24. doi: 10.1016/S0929-1393(00)00068-8

[B76] Vance E. D. Brookes P. C. Jenkinson D. S. (1987). An extraction method for measuring soil microbial biomass C. Soil Biol. Biochem. 19, 703–707. doi: 10.1016/0038-0717(87)90052-6

[B77] Varatharajan T. Dass A. Choudhary A. K. Sudhishri S. Pooniya V. Das T. K.. (2022). Integrated management enhances crop physiology and final yield in maize intercropped with blackgram in semiarid south Asia. Front. Plant Sci. 13. doi: 10.3389/fpls.2022.975569 PMC9538492 36212325

[B78] Wang Q. Liu J. Zhu H. (2018). Genetic and molecular mechanisms underlying symbiotic specificity in legume-*Rhizobium* interactions. Front. Plant Sci. 9, 313. doi: 10.3389/fpls.2018.00313 29593768 PMC5854654

[B79] Yan W. Tinker N. A. (2006). Biplot analysis of MET data: Principals and applications. Can. J. Plant Sci. 86, 623–645. doi: 10.4141/P05-169

[B80] Yusuf A. A. Abaidoo R. C. Iwuafor E. N. O. Olufajo O. O. Sanginga N. (2009). Rotation effects of grain legumes and fallow on maize yield, microbial biomass and chemical properties of an Alfisol in the Nigerian Savanna. Agric. Ecosyst. Environ. 129, 325–331. doi: 10.1016/j.agee.2008.10.007

[B81] Zahran H. H. (1999). *Rhizobium*-legume symbiosis and nitrogen fixation under severe conditions and in an arid climate. Microbiol. Mol. Biol. Rev. 63, 968–989. doi: 10.1128/MMBR.63.4.968-989.1999 10585971 PMC98982

[B82] Zhong W. H. Cai Z. C. (2007). Long-term effects of inorganic fertilizers on microbial biomass and community functional diversity in a paddy soil derived from quaternary red clay. Appl. Soil Ecol. 36, 84–91. doi: 10.1016/j.apsoil.2006.12.001

