# Supplementary material for: High-value crops’ embedded groundnut-based production systems vis-à-vis system-mode integrated nutrient management: long-term impacts on system productivity, system profitability, and soil bio-fertility indicators in semi-arid climate
Source: Front Plant Sci. 2024 Jan 4;14:1298946. doi: 10.3389/fpls.2023.1298946 (PMC10794608; doi:10.3389/fpls.2023.1298946)
Supplement: Supplementary file 1 [file DataSheet_1.pdf]

High-value crops' imbedded groundnut-based production systems vis-à-vis system-mode integrated nutrient management: Long-term impacts on system-productivity, system-profitability and soil bio-fertility indicators in semi-arid climate

Ram Swaroop Bana[1], Anil K. Choudhary[1,2*], Ravi C. Nirmal[1], Bhola Ram Kuri[1,3], Seema Sangwan[1], Samarth Godara[4], Ruchi Bansal[5], Deepak Singh[4] and D.S. Rana[1]

[1]ICAR–Indian Agricultural Research Institute, New Delhi, India–110 012
[2]ICAR-Central Potato Research Institute, Shimla, Himachal Pradesh, India–71 001
[3]ICAR-Central Arid Zone Research Institute, KVK Pali-Marvar, India–306 401
[4]ICAR–Indian Agricultural Statistics Research Institute, New Delhi, India–110 012
[5]ICAR–National Bureau of Plant Genetic Resources, New Delhi, India–110 012

***Corresponding: authors' email**: anilhpau2010@gmail.com

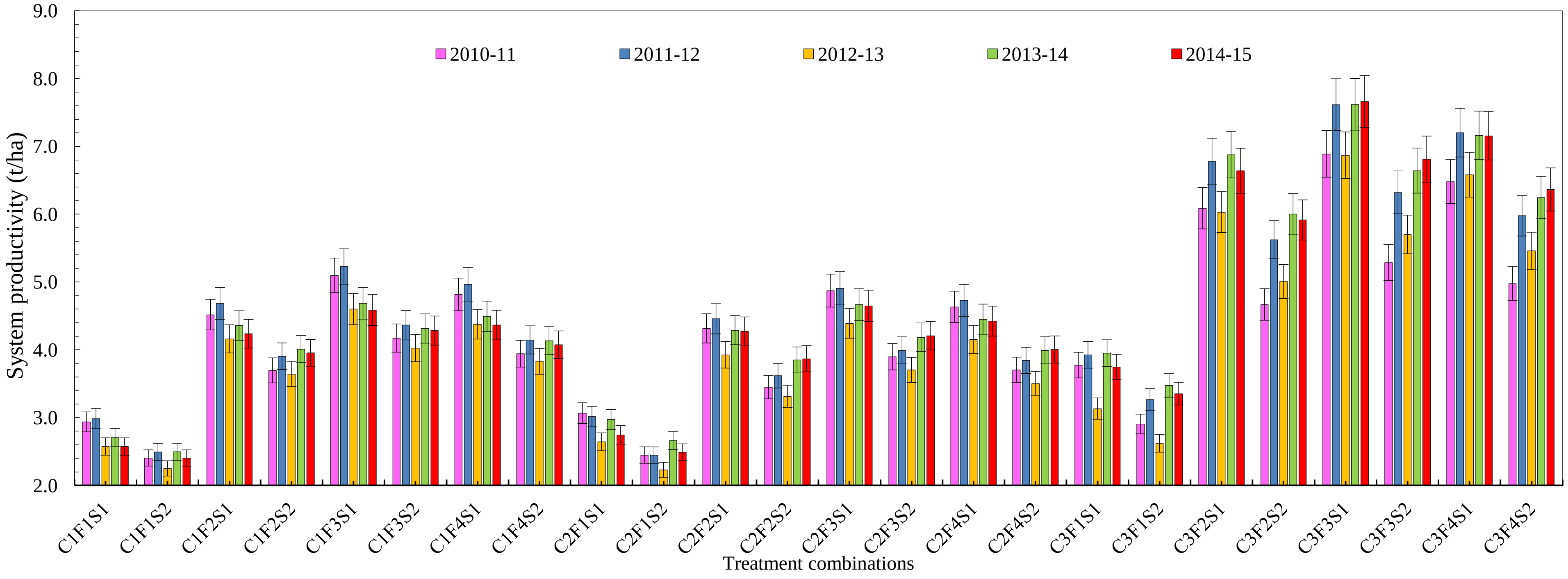

SUPPLEMENTARY FIGURE 1

Effect of GBCSs and different INM schedules on system net-returns (USD/ha). *(The vertical bars represent $LSD_{0.05}$ values).*

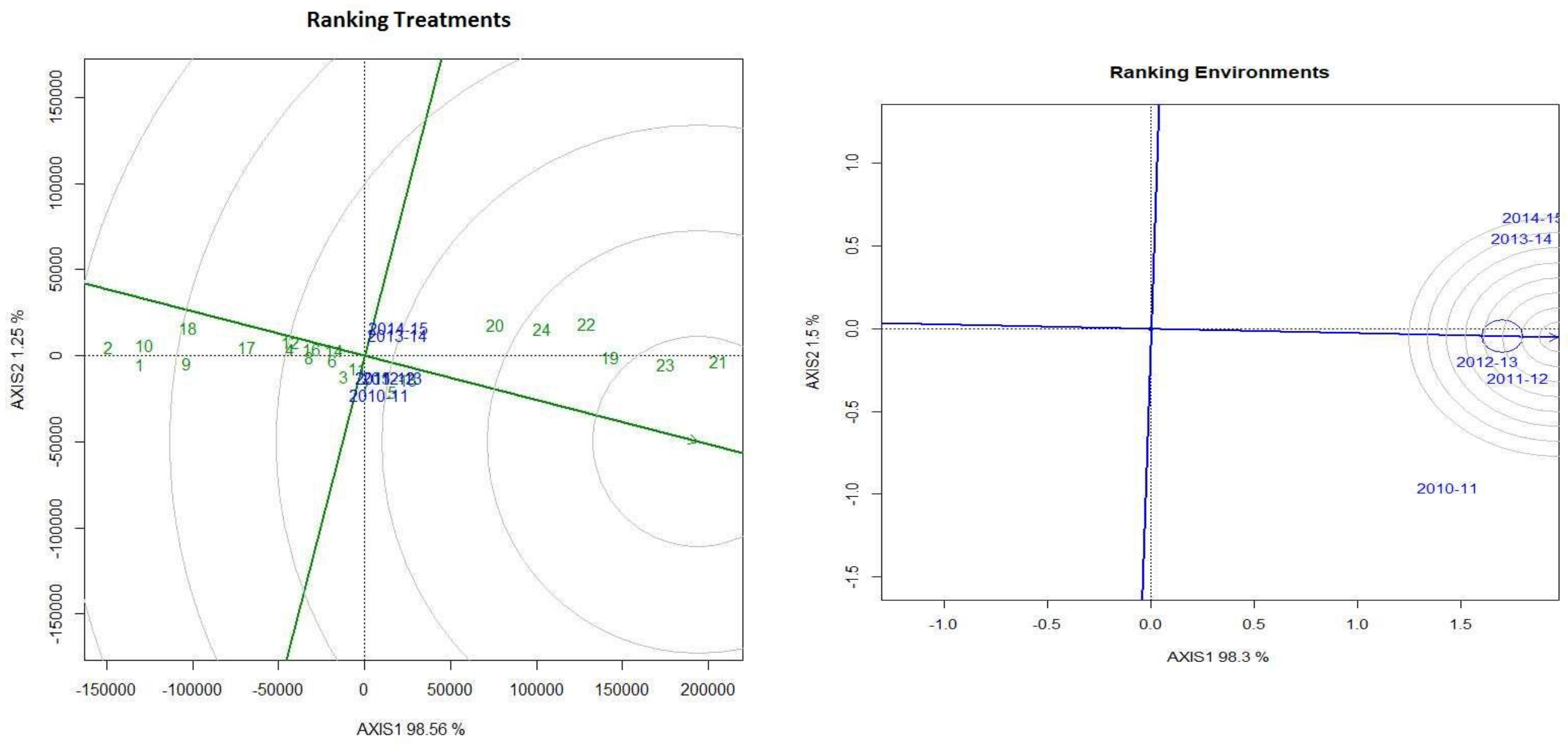

SUPPLEMENTARY FIGURE 2

GGE biplots (**A**) Ranking treatments biplot arranged the treatments in order of their performance, (**B**) Ranking environments biplot arranged the environments (i.e. years) in order of their performance. X axis: principal component 1- system yield; Y axis: principal component 2 -gross returns.
